# Forewing color pattern in Micropterigidae (Insecta: Lepidoptera): homologies between contrast boundaries, and a revised hypothesis for the origin of symmetry systems

**DOI:** 10.1186/s12862-016-0687-z

**Published:** 2016-05-26

**Authors:** Sandra R. Schachat, Richard L. Brown

**Affiliations:** Mississippi Entomological Museum, Mississippi State, MS 39762 USA; Department of Paleobiology, Smithsonian Institution, Washington, DC 20013 USA

**Keywords:** Developmental constraints, Microlepidoptera, Nymphalid groundplan, Symmetry systems

## Abstract

**Background:**

Despite the great importance of lepidopteran wing patterns in various biological disciplines, homologies between wing pattern elements in different moth and butterfly lineages are still not understood. Among other reasons, this may be due to an incomplete understanding of the relationship between color pattern and wing venation; many individual wing pattern elements have a known relationship with venation, but a framework to unite all wing pattern elements with venation is lacking. Though plesiomorphic wing veins are known to influence color patterning even when not expressed in the adult wing, most studies of wing pattern evolution have focused on derived taxa with a reduced suite of wing veins.

**Results:**

The present study aims to address this gap through an examination of Micropterigidae, a very early-diverged moth family in which all known plesiomorphic lepidopteran veins are expressed in the adult wing. The relationship between wing pattern and venation was examined in 66 species belonging to 9 genera. The relationship between venation and pattern element location, predicted based on moths in the family Tortricidae, holds for *Sabatinca* just as it does for *Micropterix*. However, the pattern elements that are lightly colored in *Micropterix* are dark in *Sabatinca*, and vice-versa. When plotted onto a hypothetical nymphalid wing in accordance with the relationship between pattern and venation discussed here, the wing pattern of *Sabatinca doroxena* very closely resembles the nymphalid groundplan.

**Conclusions:**

The color difference in pattern elements between *Micropterix* and *Sabatinca* indicates that homologies exist among the contrast boundaries that divide wing pattern elements, and that color itself is not a reliable indicator of homology. The similarity between the wing pattern of *Sabatinca doroxena* and the nymphalid groundplan suggests that the nymphalid groundplan may have originated from a *Sabatinca*-like wing pattern subjected to changes in wing shape and reduced expression of venation.

**Electronic supplementary material:**

The online version of this article (doi:10.1186/s12862-016-0687-z) contains supplementary material, which is available to authorized users.

## Background

Color pattern in the animal kingdom has been an area of intense study for well over a century [1]. Insects in the order Lepidoptera were the subject of groundbreaking research during the early years of evolutionary biology [2–5] and remain a tremendously popular system for the study of color pattern in a variety of disciplines, ranging from theoretical biology to taxonomy, developmental biology, and ecology [6–9]. However, a disproportionate number of studies of Lepidoptera – such as those cited thus far – have focused on butterflies; the evolutionary history of wing pattern in microlepidoptera is still poorly known. Due to this lack of knowledge regarding wing pattern in more early-diverging lineages, it is difficult to extrapolate findings regarding butterfly wing patterns to other lineages of Lepidoptera.

The present study aims to bridge the gap between butterflies and microlepidoptera. Here we report on wing patterns in *Sabatinca* and other genera in the family Micropterigidae. Recent studies have confirmed that Micropterigidae, along with Agathiphagidae, are the most basal living moths [10, 11]. Due to the basal phylogenetic position of Micropterigidae, commonalities between the wing patterns of butterflies and *Sabatinca* should be indicative of ancestral states for all Lepidoptera. Comparisons of the wing patterns of Micropterigidae and butterflies can be confounded by the great differences in wing size and shape in these two clades. However, a recent examination of wing pattern in *Micropterix*, another genus within the Micropterigidae, showed a consistent relationship between wing venation and color pattern [12]. This relationship with wing venation has the potential to facilitate comparisons of wing pattern in various lepidopteran lineages, as homologies among wing veins are far better understood.

**Fig. 1:**
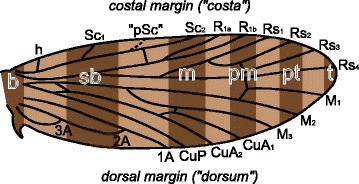
The *Micropterix* wing pattern groundplan. The forewing color pattern groundplan of *Micropterix* [12], plotted onto a hypothesized ancestral wing for Lepidoptera [15]. The vein labeled “pSc” is a known plesiomorphic wing vein for Amphiesmenoptera hypothesized by Schachat and Brown to influence color pattern. Abbreviations for other veins are as follows: h: humeral; Sc: subcosta; R: radius; Rs: radial sector; M: media; CuA: anterior cubitus; CuP: posterior cubitus; A: anal. Abbreviations for fasciae are as follows: b: basal; sb: subbasal; m: median; pm: postmedian; pt: preterminal; t: terminal

The wing pattern groundplan of *Micropterix* follows the predictions of a model based on the (far more derived) microlepidopteran family Tortricidae [13, 14]. This model is referred to as the “wing-margin” model here. In *Micropterix*, alternating light and dark brown transverse bands, or fasciae, straddle one vein each along the costal margin of the wing (Fig. 1). This interpretation relies on three veins that are not present in the adult wing of *Micropterix*. Two of these veins – h and R_1a_ – are known from other Micropterigidae such as *Sabatinca* and have been included in the most recent reconstruction of ancestral wing venation for Lepidoptera [15], and one other – a third branch of Sc, occurring here between the branches referred to as Sc_1_ and Sc_2_ – is widely known from the amphiesmenopteran fossil record [16–18] and from the basal trichopteran genus *Rhyacophila* [19]. This plesiomorphic three-branched Sc vein has also been put forth as tentative explanation for the hindwing venation of the micropterigid *Paramartyria semifasciella* [20].

In addition to the “wing-margin” model, another predictive model, proposed decades earlier by Hennig Lemche [21, 22] and referred to here as the “vein-fork” model, predicts that the basal edge of each fascia will fall along the points where veins bifurcate. The wing pattern of *Micropterix* is not consistent with the “vein-fork” model [12]. Genera such as *Sabatinca*, which have a more complete suite of plesiomorphic wing veins than *Micropterix*, are excellent candidates for further testing of the wing pattern groundplan proposed based on *Micropterix*.

### The origin of butterfly “symmetry systems”

**Fig. 2:**
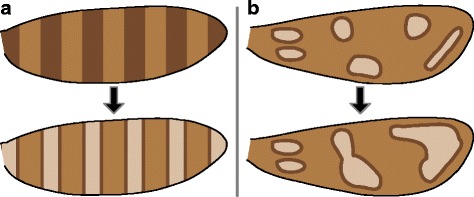
The two existing hypotheses for the origin of symmetry systems from primitive microlepidopteran wing pattern elements. **a** Lemche’s “split-band” hypothesis. **b** Nijhout’s “fused-spot” hypothesis

Butterfly wing patterns follow the “nymphalid groundplan,” which consists mainly of “symmetry systems”: nested transverse bands that run from the costal to the dorsal wing margin, symmetrical not necessarily in shape but in color [23]. Symmetry systems have been understood for nearly a century [24, 25], have undergone a revival among evolutionary and developmental biologists during recent decades [23], and remain an active area of inquiry [26]. Although symmetry systems provide the foundation for numerous publications on evolution and development, their evolutionary history remains obscure. Two hypotheses have been put forth to explain the origin of symmetry systems, both largely speculative. First, Lemche [22] hypothesized that symmetry systems originated when primitive transverse bands of a single color became bisected with another color, so that one band appears to split into two (Fig. 2a). This is called the “split-band” hypothesis here. Decades later, Nijhout [27] hypothesized that symmetry systems originated when primitive irregular spots became concentric (with a circular outline surrounding the central spot), became aligned into parallel rows running from the costal margin to the dorsal margin, and then fused into three symmetry systems (Fig. 2b). This is called the “fused-spot” hypothesis here. On the wings of moths in the families Hepialidae and Zygaenidae, concentric spots have been noted to show varying degrees of fusion [27]. However, bands that are formed by fused spots do not bear a particularly strong resemblance to nymphalid symmetry systems, and the directionality of change between spots and bands cannot yet be inferred because the necessary phylogenetic topology is still lacking.

These two hypotheses are founded on different assumptions. Lemche, after closely studying wing pattern in many families of microlepidoptera, arrived at the assumption that transverse bands are the primitive wing pattern element for Lepidoptera. Nijhout arrived at the assumption that spots, not transverse bands, are the primitive wing pattern element for Lepidoptera, because “[t]he vast majority of Panorpoid and Trichopteroid wing patterns (like those of many primitive Lepidoptera) are in fact made up of irregular spots, and insofar as these groups are sister groups of the Lepidoptera, spotted patterns are most likely to represent the p[r]imitive (plesiomorphic) condition for the Lepidoptera” [28]. However, the connection between trichopteran spotted wing patterns and the plesiomorphic character state for Lepidoptera is not entirely straightforward. In Trichoptera, spots are very small and mainly occur along the wing margin. But in the most basal moths that have spotted wing patterns – Neopseustidae, Hepialidae, and, to a certain extent, Lophocoronidae and Eriocraniidae – spots are much larger and occur throughout the entire wing. Also, in Trichoptera as in all other Panorpoidea aside from Lepidoptera, color pattern is most often associated with the wing membrane, but in Lepidoptera, color pattern rarely occurs on the wing membrane and is nearly always associated with wing scales. The role of gene expression in wing pattern development remains largely unexplored in microlepidoptera and in Trichoptera; theoretically, the same genes could influence the development of color pattern in all cells on the wing, regardless of whether a cell ultimately differentiates into an epithelial cell or a scale. But because color pattern elements on the wings of Trichoptera and Lepidoptera are very different in appearance and occur in different anatomical structures – membrane versus scales – proposed homology of color pattern elements between these two orders remains somewhat dubious.

The two mutually exclusive hypotheses for the origin of symmetry systems rely on conflicting assumptions; a test of these assumptions would be a first step toward rejecting one, or perhaps ultimately both, of these hypotheses. In addition to the primitive state for lepidopteran wing pattern elements, another question must be resolved in order to reconcile microlepidopteran wing pattern with the origin of symmetry systems: the influence of wing venation on wing pattern development. In Lepidoptera with symmetry systems, such as butterflies, certain types of wing pattern elements – such as venous stripes – are unquestionably dependent on venation [23]. Other pattern elements, such as melanic band pattern elements, are not so obviously dependent on venation and may develop even if a species’ typical suite of venation is not expressed in the adult wing [29]. However, observations over many decades have confirmed that plesiomorphic veins can continue to influence the development of butterfly wing pattern elements, such as eyespots, even if the veins are not expressed in the adult wing [24, 30], so the presence of a wing pattern element in the absence of expressed corresponding venation does not necessarily indicate that wing venation is irrelevant to that pattern element. Because many plesiomorphic lepidopteran wing veins are not distinguishable in the adult wings of butterflies and other higher Lepidoptera, either due to fusion or lack of expression, the relationship between venation and color patterning is especially difficult to deduce in these taxa, and has been granted little consideration.

### Primitive lepidopteran wing venation

Though we do know with absolute certainty that plesiomorphic wing veins can influence color pattern elements (such as eyespots) even when not expressed, the relevant groundplan of primitive lepidopteran wing venation remains less certain. Because lepidopteran color patterns are unique in that they arise from scales, a reasonable working hypothesis is that the veins with potential to influence the development of color pattern are those that were present in the ancestral lineage in which scales first originated, regardless of whether these primitive scales expressed color. However, identification of this lineage, and the wing veins that it possessed, is hindered by the nature of the lepidopteran fossil record. The timing of the split between Trichoptera and Lepidoptera is unknown; the earliest definitive Trichoptera and Lepidoptera both date to the Mesozoic, but the recent finding of putative caddisfly cases from the early Permian would move this split much farther into the past [31]. Moths have a remarkably poor fossil record [32] and putative stem-group fossils are plagued by taxonomic uncertainty [33]. In the earliest known fossil of a true moth, *Archaeolepis mane*, only one branch of the Sc vein is visible [34, 35]. But early-diverging moths such as Micropterigidae overwhelmingly possess a two-branched Sc vein, and because a multi-branched Sc vein is the plesiomorphic character state for ancestral Amphiesmenoptera [16–18], *A. mane* is highly unlikely to represent the ancestral state for lepidopteran wing venation.

Other Jurassic fossil moths offer limited additional information about ancestral wing venation. New discoveries are very rare [15], and fossils previously assigned to the extinct trichopteran family Necrotauliidae have been shifted to Lepidoptera on the basis of wing venation – more specifically, a 3-branched medial vein [36]. Unsurprisingly in light of the fact that assignment of Jurassic amphiesmenopteran fossils to Lepidoptera depends largely on similarities with venation in extant moths, the most recent hypothesis for primitive lepidopteran venation [15] bears a striking resemblance to wing venation in Micropterigidae such as *Sabatinca*. There is reason to doubt that this hypothesis is complete: it contains a three-branched M vein, as is found in *Sabatinca* and other Micropterigidae, but the presence of a four-branched M vein in Permotrichoptera [17, 18], Mesozoic caddisflies [37], extant caddisflies [19], and the extant lepidopteran family Agathiphagidae [38] – recently shown to belong to the earliest-diverging branch of Lepidoptera, alongside Micropterigidae [10, 11] – suggests that more veins may need to be added to the reconstruction of primitive moth venation. Given the paucity of data available to inform hypotheses of primitive lepidopteran wing venation, the possibility certainly exists that additional wing veins known from other Amphiesmenoptera may have also been present in early moths, and may therefore continue to influence the development of color patterns in extant Lepidoptera.

### Micropterigidae: systematics and wing pattern morphology

**Fig. 3:**
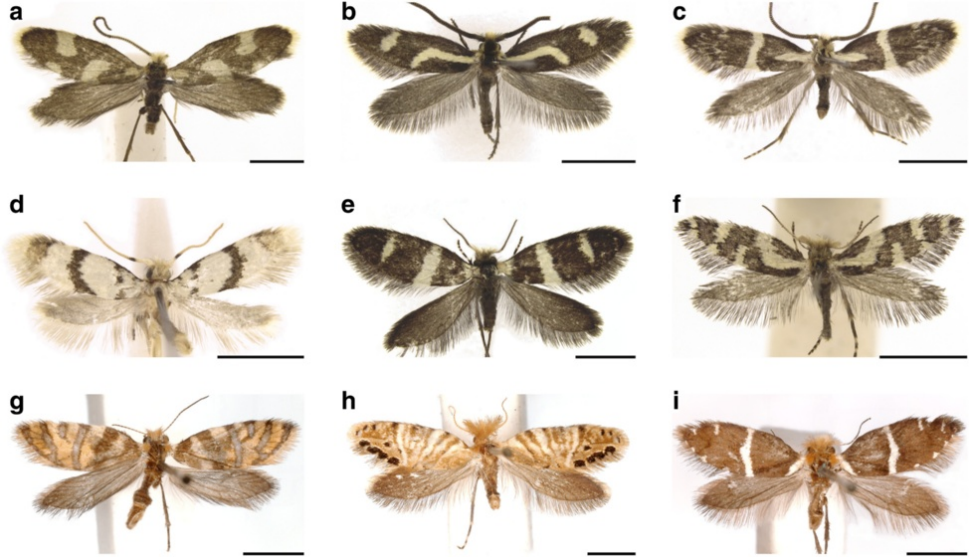
Representatives of various taxa examined for the present study. **a** *Epimartyria pardella*. **b** *Tasmantrix lunaris*. **c** *Tasmantrix nigrocornis*. **d** *Aureopterix sterops*. **e** *Austromartyria porphyrodes*. **f** *Agrionympha capensis*. **g** *Sabatinca aurella*. **h** *Sabatinca calliarcha*. **i** *Sabatinca ianthina*. All scale bars represent 2 mm

Micropterigidae are small moths with varied wing patterns (Fig. 3). Modern molecular studies have confirmed that Micropterigidae, along with Agathiphagidae, are the most basal living moths [10, 11]. Relationships within the Micropterigidae were recently explored in a molecular “preliminary phylogeny” based on the COI gene, which included 47 species of *Sabatinca*, 12 other micropterigid genera, and trichopteran outgroups [39]. Results of the molecular analysis were consistent with previous hypotheses based on morphology alone. The deepest split within the Micropterigidae has resulted in two biogeographically distinct clades: one in the Southern Hemisphere (Gondwanan) and one in the Northern Hemisphere (Laurasian). This Gondwanan-Laurasian split was also found in another recent study that included fewer micropterigid taxa but more comprehensive gene sampling [11]. The Laurasian clade consists of the early-diverging genus *Micropterix*, whose wing pattern has already been examined [12]; *Epimartyria*, a genus with three species, two of which have wing patterns comprised of large, light brown spots against a dark brown ground color [40]; and various genera whose wings are covered entirely is dark brown, purplish, or reddish scales [20]. The Gondwanan clade contains many genera whose wing patterns include two or more colors, and is the focus of the present study. This clade contains two lineages: *Sabatinca* plus the “southern sabatincoid” lineage, which is dispersed throughout the Southern Hemisphere and includes *Austromartyria*, *Hypomartyria*, and *Agrionympha*; and the second, far less diverse “Australian group” which is restricted to Australia, New Zealand, and New Caledonia and is dominated by *Tasmantrix* and also includes *Aureopterix*, *Zealandopterix*, and *Nannopterix*. The terms “southern sabatincoid” and “Australian group” are used here in accordance with previous contributions [39].

Studies of micropterigid wing scales have found that they are always internally “fused” and therefore lack a cavity to hold pigment sacs [41]. Photos and written descriptions show that micropterigid wing scales are often iridescent [39, 42, 43].

### Terminology

Terminology is used as follows. A “wing pattern element” formed by two or more adjacent wing scales of the same color, and can take the form of a spot, band, patch, etc., and the term “band” is used as shorthand for “transverse band,” which is a band that runs more or less between the costal and dorsal margins of the wing. The term “fascia” is rarely used here; this term has recently been subject to varying interpretations regarding wing pattern in Micropterigidae, having been applied to both light bands [43, 44] and to dark bands [12]. The term “band,” as used here, encompasses both interpretations of “fascia.” Similarly, the term “ground color” is avoided here because this term usually signifies the color that covers the greatest amount of wing surface area, but this can vary between very light and very dark brown even among closely related species within the same genus. For the sake of simplicity, the term “color” is applied broadly, to encompass all distinguishable colors, shades, tones, and tints. Therefore, white, silver, blue, brown, and black are discussed as “colors,” and different tints, tones, or shades – for example, light and dark shades of brown – are considered to be separate colors. The use of the term “color” here does not discriminate between structural colors and those derived from pigments. Nomenclature for wing venation (Fig. 1) mainly follows Wootton [45], with the exception of the humeral vein (“h”). The wing veins referred to with conventional nomenclature are those that are visible in the adult wing of *Sabatinca*. Schachat and Brown hypothesized that a third branch of Sc, known to be plesiomorphic for Amphiesmenoptera, plays a key role in micropterigid wing pattern; this hypothesized vein is referred to here as “pSc,” for plesiomorphic Sc (Fig. 1). Many species from New Caledonia are referred to here with numbers (“*Sabatinca* spp. 43, 20, 47, 29, 46, and 11,” etc.); these are the same numbers that were used to refer to undescribed species included in the recent *Sabatinca* phylogeny [39].

## Results

### Forewing pattern in New Zealand *Sabatinca*

**Fig. 4:**
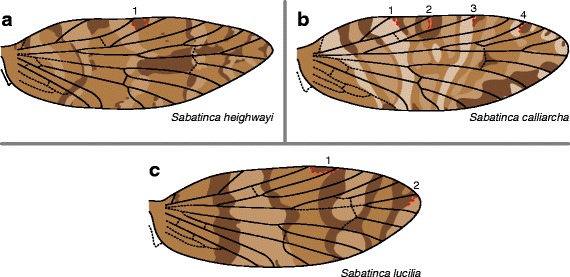
Wing pattern of the most basal *Sabatinca* species, which occur in New Zealand and belong to the *calliarcha* group. **a** *S. heighwayi*.** b** *S. calliarcha*. **c** *S. lucilia*

In the *Sabatinca* clade shown to be the most basal in the genus, called the “*calliarcha* group” [39], wing pattern consists of either three or four different colors in each species (Fig. 4). *Sabatinca lucilia* has the simplest wing pattern in this group, with a small, dark pattern element straddling the humeral vein; a very light-colored, uninterrupted band straddling Sc_1_ at the costa, surrounded by a dark band on each side; another very light band straddling R_1b_ at the costa, also surrounded by a dark band on each side but interrupted by a dark patch connecting the two dark bands; and one last very light band, straddling Rs_4_ and M_1_ along the dorsum and sometimes Rs_3_ along the costa, also bordered by a dark band along its basal edge (Fig. 4c). *Sabatinca heighwayi* has a somewhat similar pattern in that it includes very light bands surrounded by dark bands, but the bands on *Sabatinca heighwayi* are more numerous and not as wide (Fig. 4a). The wing pattern of *Sabatinca calliarcha* is more complex, with four colors and a number of variations (Fig. 4b). Some transverse bands are connected by patches of the same color that run along the proximo-distal axis of the wing. Concentric spots occur near the apex of the wing: on the costal margin these are comprised of small, dark intravenular patches surrounded by a rim of very light scales, with the opposite arrangement on the dorsal margin.

**Fig. 5:**
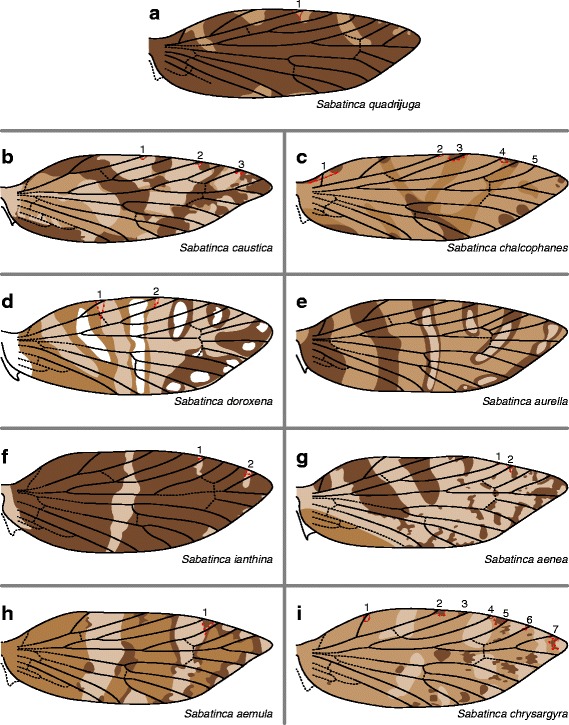
Wing pattern of the majority of New Zealand *Sabatinca* species sampled, which belong to the *chrysargyra* group. **a** *S. quadrijuga*. **b** *S. caustica*. **c** *S. chalcophanes*. **d** *S. doroxena*. **e** *S. aurella*. **f** *S. ianthina*. **g** *S. aenea*. **h** *S. aemula*. **i** *S. chrysargyra*

In the “*chrysargrya* group” – the clade that includes the majority of New Zealand *Sabatinca* species sampled – the fasciate wing pattern of *S. aurella* is the most straightforward (Fig. 5e). Dark and medium brown wing pattern elements alternate along the wing; the four distal-most dark pattern elements contain areas of light scales in the center, but these light scales never interrupt the contiguous dark border. At and beyond the terminal Sc branch, the dark and medium wing pattern elements each straddle one vein along the costal margin. The wing pattern of *Sabatinca doroxena* is very similar to that of *S. aurella*, except that the location of certain pattern elements varies slightly between individuals, and the pattern includes four colors instead of three (Fig. 5d). The wing pattern of *Sabatinca aenea* (Fig. 5g) includes many small spots and therefore is not strictly “fasciate,” particularly along the apical half of the wing; nevertheless, it is similar to that of *S. aurella* in that, with a single exception, the largest of the dark pattern elements straddle/abut alternating wing veins along the costal margin. However, wing pattern in this species is quite variable, and in certain specimens, pattern elements – some extremely small – straddle every single vein beyond Sc, instead of occurring in an alternating pattern.

The wing pattern of *Sabatinca aemula* (Fig. 5h) is similar to that of *S. aurella* in that the lightest scales form transverse markings that are bordered by the darkest scales on the wing. Two major differences between *Sabatinca aemula* and *S. aurella* are immediately apparent: firstly, the wing pattern of *S. aemula* is not entirely fasciate, as the darkest scales often form spots, and secondly, medium-colored scales straddle alternating veins along the costa of *S. aurella* but straddle only one vein, h, along the costa of *S. aemula*. The wing pattern of *Sabatinca chrysargyra* is broadly similar to that of *S. aemula* in terms of the positioning of pattern elements relative to veins along the costa, but contains spots of varying sizes instead of any discernible fasciae (Fig. 5i). In *Sabatinca chrysargyra*, unlike *S. aurella* and *S. aemula*, the darkest pattern elements are spots and do not occur adjacent to the lightest pattern elements.

The wing patterns of other *Sabatinca* species in the “*chrysargyra* group” do not lend themselves as obviously to comparison with the wing pattern of *S. aurella*, and are discussed in order of complexity as follows. In *Sabatinca ianthina*, the predominance of dark wing pattern elements is such that dark scales straddle every single vein along the costa (Fig. 5f). *Sabatinca quadrijuga* also has a wing pattern that consists overwhelmingly of dark scales; certain lighter wing pattern elements do straddle veins at the costa, but this occurs only at the h and Sc veins (Fig. 5a). *Sabatinca caustica* and *S. chalcophanes* share a banding pattern in which fasciae converge toward the middle of the dorsum (Fig. 5b, c). In both species, wing pattern is quite variable at the costal margin of the wing and all veins that reach the costa, including the humeral vein, are surrounded by dark scales in at least some specimens.

**Fig. 6:**
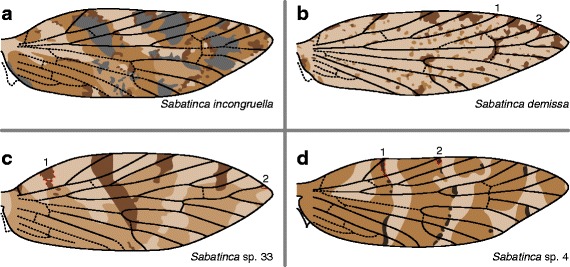
Wing pattern of the earliest-diverging species of the *incongruella* group, from New Zealand and New Caledonia. **a** *S. incongruella*. **b** *S. demissa*. **c** *S*. sp. 33. **d** *S*. sp. 4

The wing patterns of *Sabatinca incongruella* and *S. demissa*, the only two New Zealand species that belong to the “*incongruella* group,” do not consist exclusively of fasciae or spots. In *S. incongruella*, the pattern consists of four colors (Fig. 6a). Fasciae have very jagged edges and spots occur toward the dorsum. In *Sabatinca demissa* (Fig. 6b), large, dark spots occur at the points where veins meet the costa and where veins bifurcate; all veins along the costa (except for the humeral vein) are surrounded by dark scales in at least some individuals. Smaller spots occur elsewhere on the wing and are usually much lighter in color.

### Forewing pattern in New Caledonia *Sabatinca*

The two New Caledonian species shown to be most basal, *Sabatinca* spp. 33 and 4, have somewhat fasciate wing patterns consisting of three colors. In *Sabatinca* sp. 33 (Fig. 6c), only two the lightest and darkest colors reach the costa. The darkest brown straddles the humeral vein, and then alternating veins: Sc_1_, R_1a_, Rs_1_, and sometimes Rs_3_. In *Sabatinca* sp. 4, all three colors reach the costa (Fig. 6d). The main transverse bands alternate between light and medium brown, with small dark brown spots and bands appearing at the basal edge of the light bands.

**Fig. 7:**
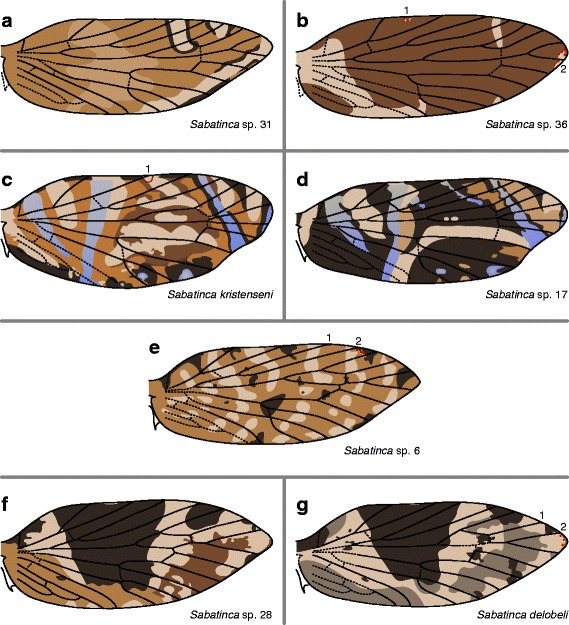
Wing pattern in the *incongruella* group from New Caledonia, continued. **a** *S*. sp. 31. **b** *S*. sp. 36. **c** *S. kristenseni*. **d** *S*. sp. 17. **e** *S*. sp. 6. **f** *S*. sp. 28. **g** *S. delobeli*

*Sabatinca* sp. 31 (Fig. 7a) has two light bands, outlined by very dark scales, straddling R_1a_ and Rs_1_ at the costa. Another light band, with no dark outline, abuts Rs_3_ and straddles Rs_4_. *Sabatinca* sp. 36 (Fig. 7b) has a wing pattern that consists primarily of dark scales; lighter wing pattern elements are few and small. One light pattern element straddles Rs_4_ in most specimens, another reaches the costa between R_1a_ and R_1b_, and one more occasionally appears in the “pSc” region of the costa, but no light pattern elements straddle any visible veins along the costa. In *Sabatinca kristenseni* and sp. 17 (Fig. 7c, d), veins are often abutted by two wing pattern elements at the costa. In *Sabatinca kristenseni*, Sc_2_ and Rs_2_ are straddled by light pattern elements; R_1a_, R_1b_, and Rs_1_ are straddled by dark pattern elements; and Rs_4_ is straddled by a blue pattern element. In *Sabatinca* sp. 17, Rs_1_ is surrounded by a dark pattern element, Rs_2_ is surrounded by a light pattern element, and Rs_4_ is surrounded by a blue pattern element. In *Sabatinca* sp. 6, dark pattern elements always straddle veins Sc_1_, Sc_2_, Rs_1_, and Rs_3_, and sometimes straddle R_1a_ and R_1b_, along the costa (Fig. 7e). Five very light bands also reach the costa but these never straddle, and rarely abut, any veins. In *Sabatinca delobeli* and sp. 28 (Fig. 7f, g), dark pattern elements straddle all veins except for Rs_3_ at the costa.

**Fig. 8:**
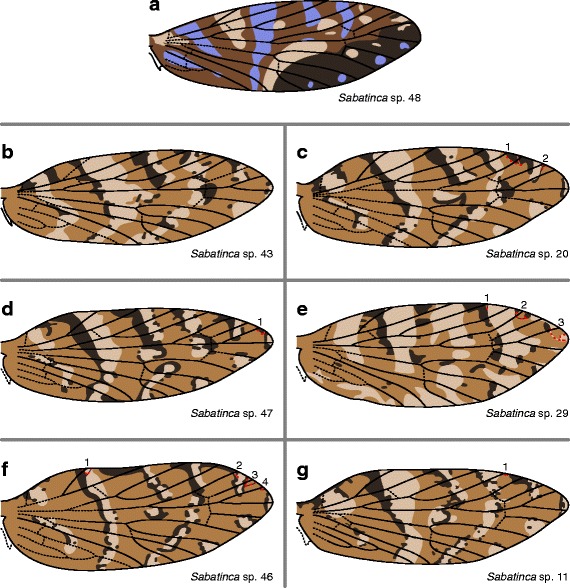
Wing pattern in the *incongruella* group from New Caledonia, continued. **a** *S*. sp. 48. **b** *S*. sp. 43. **c** *S*. sp. 20. **d** *S* *.* sp. 47. **e** *S*. sp. 29. **f** *S*. sp. 46. **g** *S*. sp. 11

The wing pattern of *Sabatinca* sp. 48 includes four colors: beige, pale blue, and two shades of dark brown (Fig. 8a). The Sc_1_ vein abuts a dark brown pattern element, and all other veins are straddled by dark brown pattern elements along the costa. *Sabatinca* spp. 43, 20, 47, 29, 46, and 11 (Fig. 8b-g) all have similar wing patterns – somewhat reminiscent of those of *Sabatinca aemula*, *S. heighwayi*, and *S*. sp. 4 – with light brown bands against a medium background bordered, most often on the basal edge, by small dark stripes and spots. In these six species, light bands surround/abut Sc_1_ and Sc_2_. In *Sabatinca* sp. 29 (Fig. 8e), R_1a_, Rs_1_, and Rs_3_ are most often surrounded by medium colored scales, and R_1b_ and Rs_2_ are surrounded by light scales. In the other five species in this clade, dark scales nearly always surround/abut R_1a_, R_1b_, and Rs_1_ at the costa, and medium scales usually surround/abut Rs_2_ and Rs_3_.

**Fig. 9:**
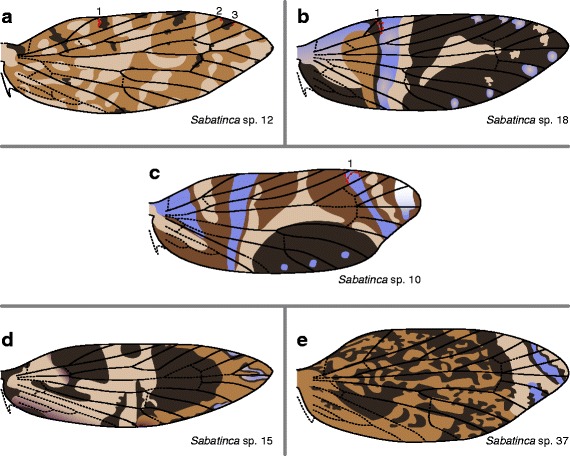
Wing pattern in the *incongruella* group from New Caledonia, continued. **a** *S*. sp. 12. **b** *S*. sp. 18. **c** *S*. sp. 10. **d** *S*. sp. 15. **e** *S*. sp. 37

In *Sabatinca* sp. 12 (Fig. 9a), all veins that terminate along the costa are surrounded by dark scales in some or all of the specimens examined. As in *Sabatinca* sp. 6 (Fig. 7e), various light bands occur along the costa, but never straddle or abut any veins. In *Sabatinca* sp. 18 (Fig. 9b), Sc_1_ usually abuts a dark wing pattern element, and dark scales surround all other veins at the costa. *Sabatinca* sp. 10 (Fig. 9c) has a wing pattern broadly similar to that of *Sabatinca* sp. 18, but Sc_1_, Rs_3_, and sometimes R_1b_ are surrounded by bluish scales. *Sabatinca* spp. 15 and 37 (Fig. 9d, e) have wing patterns unlike those of any other *Sabatinca* species; these two color patterns are quite different from each other, with the exception of small, bluish wing pattern elements toward the wing apex. In *Sabatinca* sp. 15 (Fig. 9d), dark scales surround Sc_2_, Rs_1_, and Rs_2_ at the costa; all other veins are surrounded by scales in one of two shades of lighter brown. In *Sabatinca* sp. 37 (Fig. 9e), Sc_1_ is abutted basally by dark scales and apically by light scales, and beyond this vein, dark and light pattern elements occur in an alternating fashion from Sc_2_ (surrounded by light scales) through Rs_2_. At the wing apex, Rs_3_ violates this pattern of alternation because it is surrounded by the same light pattern element as Rs_2_.

**Fig. 10:**
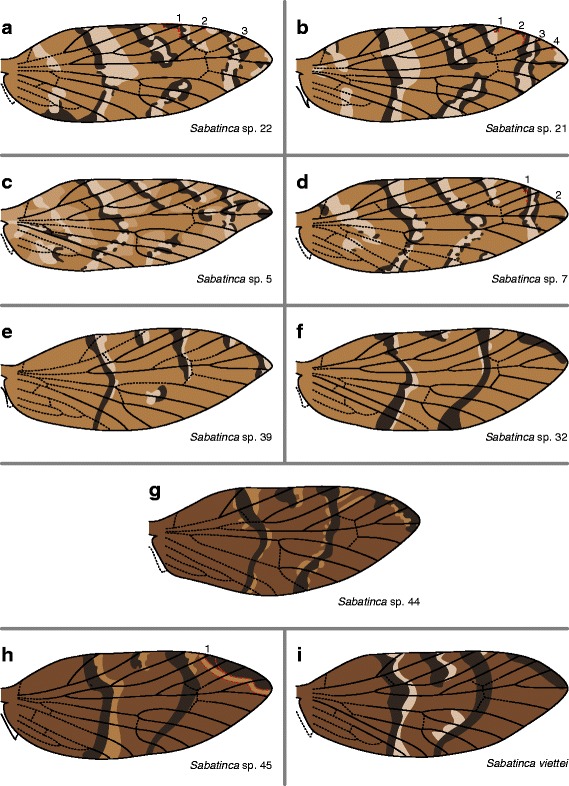
Wing pattern in the *incongruella* group from New Caledonia, continued. **a** *S*. sp. 22. **b** *S*. sp. 21. **c** *S*. sp. 5. **d** *S*. sp. 7. **e** *S*. sp. 39. **f** *S*. sp. 32. **g** *S*. sp. 44. **h** *S*. sp. 45. **i** *S. viettei*

Figure 10 shows wing pattern in two well-supported clades: the sp. 22 clade, consisting of *Sabatinca* spp. 22, 21, 5, and 7; and the *viettei* clade, consisting of *Sabatinca* spp. 44, 39, 32, 45, and *viettei* [39]. Species in the sp. 22 clade have wing patterns comprised of light bands, with each light band bordered with small dark bands and spots, mostly along the basal edge (Fig. 10a-d), very reminiscent of the wing patterns found in *Sabatinca* spp. 43, 20, 47, 46, and 11 (Fig. 8). In all species of the sp. 22 clade, light bands straddle/abut Sc_1_ and Sc_2_, dark scales straddle/abut R_1a_ and Rs_1_, and medium scales straddle/abut R_1b_ and Rs_2_. In *Sabatinca* spp. 21 and 7 (Fig. 10b, d), dark spots sometimes straddle Rs_3_. The *viettei* clade also contains wing patterns with dark bands occurring basally to light bands (Fig. 10e-i), but whereas the sp. 22 clade contains many wing patterns in which light bands are surrounded by dark pattern elements on both sides, the opposite is true for the *viettei* clade: light spots sometimes appear basally to the dark bands. However, the relationship between color pattern and venation along the costa is very similar in the sp. 22 and *viettei* clades: in the *viettei* clade as in the sp. 22 clade, light bands straddle/abut Sc_1_ and Sc_2_, dark bands straddle/abut R_1a_ and Rs_1_, and medium scales straddle/abut R_1b_. In the *viettei* clade, unlike the sp. 22 clade, a dark band always straddles Rs_3_. In *Sabatinca* sp. 39 (Fig. 10e), medium scales straddle Rs_2_; in all other species in the *viettei* clade, a single band straddles Rs_1_, Rs_2,_ and Rs_3_.

### Forewing pattern in other Micropterigidae

**Fig. 11:**
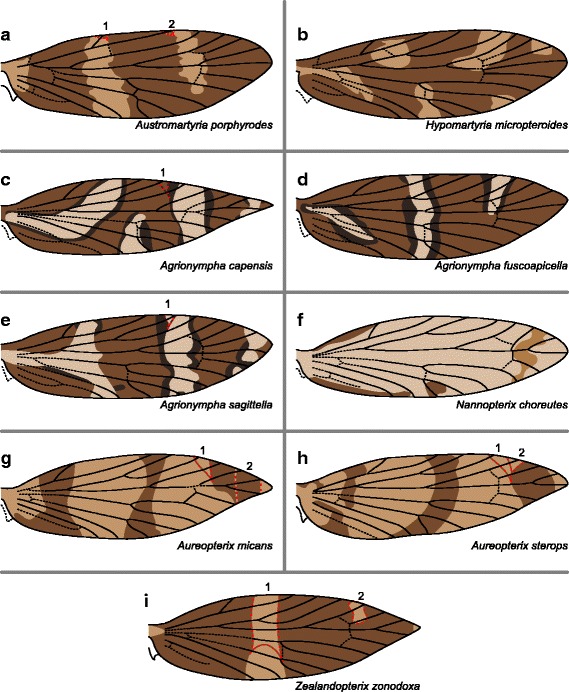
Wing pattern in Micropterigidae other than *Sabatinca*, *Tasmantrix*, *Micropterix*, and *Epimartyria*. **a** *Austromartyria porphyrodes*. **b** *Hypomartyria micropteroides*. **c** *Agrionympha capensis*. **d** *Agrionympha fuscoapicella*. **e** *Agrionympha sagittella*. **f** *Nannopterix choreutes*. **g** *Aueropterix micans*. **h** *Aureopterix sterops*. **i** *Zealandopterix zonodoxa*

In genera other than *Sabatinca*, forewing pattern is simpler, consisting of only two or three shades of brown and usually with fewer wing pattern elements due to the absence of numerous dark spots. Along the costa of *Austromartyria porphyrodes* (Fig. 11a), light bands sometimes straddle Sc_1_ and Sc_2_; dark bands straddle all other veins. In *Hypomartyria micropteroides* (Fig. 11b), one light band nearly reaches the costa between Sc_1_ and Sc_2_, another straddles R_1b_, and one more abuts Rs_2_ and straddles Rs_3_. In the three *Agrionympha* species examined – *A. capensis*, *A. fuscoapicella*, and *A. sagittella* (Fig. 11c-e) – light bands are surrounded on either side by thinner, very dark bands. As in *Hypomartyria micropteroides*, one light band reaches the costa between Sc_1_ and Sc_2_. Another light band straddles R_1a_ in *Agrionympha sagittella*, and R_1b_ in *A. capensis* and *A. fuscoapicella*. In *Agrionympha capensis* and *A. sagittella*, a third light band straddles Rs_3_; this band is absent in *A. fuscoapicella*.

In *Nannopterix choreutes* (Fig. 11f), a dark band abuts the basal edge of Sc_1_ at the costa and a medium band straddles Rs_2_. In *Aureopterix micans* (Fig. 11g), dark bands straddle Sc_1_, Sc_2_, and Rs_1_ at the costa. Sometimes the band that straddles Rs_1_ also straddles Rs_2_ and Rs_3_, and, less often, R_1b_. In *Aureopterix sterops* (Fig. 11h), dark bands consistently straddle Sc_2_ and Rs_2_ at the costa; the band that straddles Rs_2_ sometimes extends to Rs_1_ and R_1b_. In *Zealandopterix zonodoxa* (Fig. 11i), the only light wing pattern element that consistently reaches the costal margin of the wing is a small spot that occurs at the apex and does not straddle any veins; in some specimens, one light band occurs at the “pSc” area of the costa between Sc_1_ and Sc_2_ and another light band abuts Rs_1_.

**Fig. 12:**
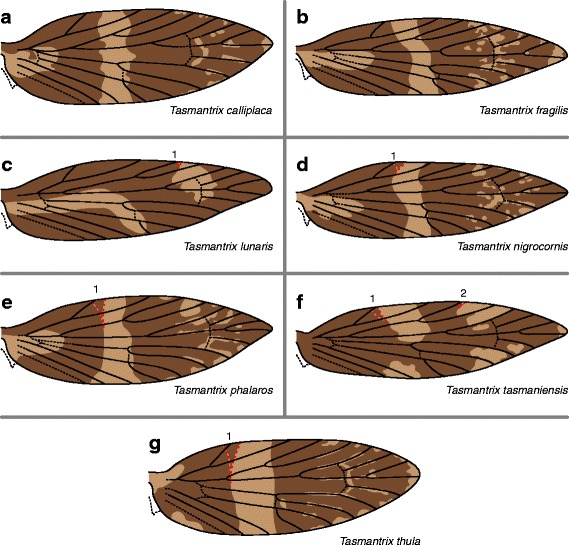
Wing pattern in *Tasmantrix*. **a** *T. calliplaca*. **b** *T. fragilis*. **c** *T. lunaris*. **d** *T. nigrocornis*. **e** *T. phalaros*. **f** *T. tasmaniensis*. **g** *T. thula*

Wing pattern in *Tasmantrix* consists of two shades of brown (Fig. 12). In all species examined here except *Tasmantrix lunaris*, a light band reaches the costa between Sc_1_ and Sc_2_; in *T. phalaros*, *T. tasmaniensis*, and *T. thula*, this band sometimes straddles Sc_1_ as well. Another light band straddles R_1b_ in *Tasmantrix tasmaniensis* and almost reaches this vein in *T. lunaris*, sometimes straddling R_1a_ in both species. In all other species – in which a band does not straddle R_1a_ or R_1b_ – small spots occur along this area of the costa but never straddle any veins. A light pattern element straddles Rs_4_ in *Tasmantrix calliplaca*, *T. tasmaniensis*, and *T. thula*, nearly abutting this vein in *T. fragilis*.

**Fig. 13:**
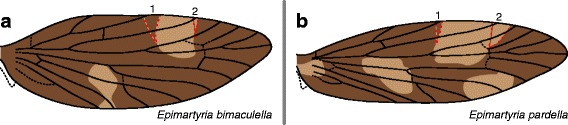
Wing pattern in *Epimartyria*. **a** *E. bimaculella*. **b** *E. pardella*

Lastly, in *Epimartyria* (Fig. 13) – the only genus examined here that belongs to the Laurasian, Northern Hemisphere clade [39] – both species, *E. bimaculella* and *E. pardella*, have the same wing pattern along the costa: a single light pattern element usually straddles R_1a_ and occasionally straddles Sc_2_.

## Discussion

### A revised hypothesis for the origin of symmetry systems

The wing patterns of certain *Sabatinca* species illustrate a straightforward mechanism though which symmetry systems could have originated. This observation has led us to revise Lemche’s original “split-band” hypothesis for the origin of symmetry systems so that it includes aspects of the “wing-margin” model.

**Fig. 14:**
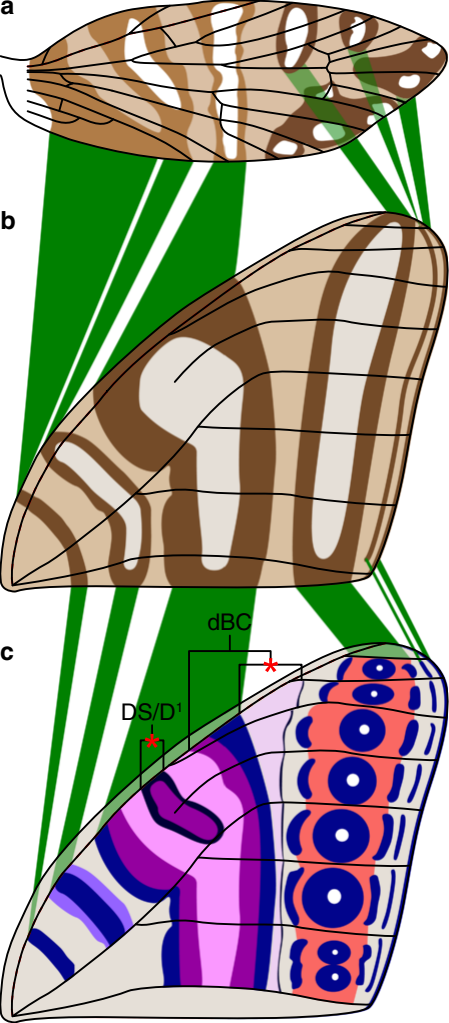
Comparison of the wing pattern of *Sabatinca doroxena* with the nymphalid groundplan. **a** The observed wing pattern of *Sabatinca doroxena*. **b** The wing pattern of *Sabatinca doroxena* plotted onto a typical wing venation groundplan for Nymphalidae, preserving the relationship between venation and pattern outlined by the “wing-margin” model. **c** The nymphalid groundplan [26, 46], with the discal spot/Discalis 1 (DS/D^1^) and the distal band of the central symmetry system (dBC) labeled and indicated with red asterisks; these two features of the nymphalid groundplan have no counterpart in the wing pattern of *Sabatinca doroxena*

Because micropterigid wing pattern is consistent with Lemche’s assumption that transverse bands, not spots, are the primitive wing pattern element for Lepidoptera, Nijhout’s “fused-spot” hypothesis appears to be an unlikely explanation for the origin of symmetry systems. In contrast, the wing patterns of *Sabatinca doroxena* and *S. aurella* are entirely consistent with Lemche’s “split-band” hypothesis, which requires a band that was originally of a single color to take on another color in its center while remaining self-symmetrical. In both species, the dark band that straddles the humeral vein is comprised of a single color, but the bands that straddle “pSc”, R_1a_, Rs_1_, and Rs_3_ are all bisected by a much lighter color, exactly as predicted by Lemche. A projection of the wing pattern of *Sabatinca doroxena* onto a typical nymphalid wing venation groundplan further supports the “split-band” hypothesis, given that the projection is carried out in accordance with the “wing-margin” model (Fig. 14).

Because micropterigid and nymphalid wings are so different in size, shape, and venation, the wing pattern of *Sabatinca doroxena* could be projected onto a nymphalid wing in any number of ways. However, the exacting predictions of the “wing-margin” model allow this to occur in only one way. In *Sabatinca doroxena*, the basal half of the nearly-split band that straddles the humeral vein is comprised of a single color, the apical half is bisected by a lighter color, and one more band – again, bisected by a lighter color – reaches the margin of the wing basal to the terminal branch of the subcostal vein. According to the nymphalid groundplan, three such bands – a unicolorous band at the base of the wing, followed by two symmetry systems – reach the costa before Sc terminates. On the wing of *Sabatinca doroxena*, three additional concentric, two-color bands occur between the terminal branch of Sc and the terminal branch of Rs. However, because Sc terminates so close to the apex in Nymphalidae, the nymphalid wing simply does not contain sufficient space for three symmetry systems beyond Sc – much in the same way that the pattern element straddling Rs_4_ in *Micropterix* originates along the costa and develops into a band, and is called the “terminal fascia,” but the pattern element straddling this same vein in Tortricidae does not occur along the costa of the wing and can only exist as a spot [12]. And so in Nymphalidae, there is only space for one symmetry system beyond Sc; two very thin unicolorous bands appear between this symmetry system and the termen of the wing, such that the total number of wing pattern elements beyond Sc is the same in Nymphalidae as it is in *Sabatinca doroxena*; the fact that the two terminal wing pattern elements in Nymphalidae do not resemble those in *Sabatinca doroxena* is a necessity according to the “wing-margin” model due to differences in wing shape between these two taxa (Fig. 14).

Most features of the nymphalid groundplan have a corresponding pattern element in *Sabatinca doroxena* and *S. aurella*. The only nymphalid groundplan features not accounted for in the wing patterns of *S. doroxena* and *S. aurella* are the discal spot (“DS” or “DI”) and the distal portion of the distal band of the central symmetry system (“dBC”), a feature that is illustrated as a discrete entity by some authors [26] but treated simply as the distal margin of the central symmetry system by other authors [46]. The discal spot could have arisen if the central symmetry system, corresponding to the pattern element that located in the “pSc” area of the wing in *Sabatinca*, originated from a band that hypertrophied not once but twice. A discrete, visible “dBC” pattern element, marked with an asterisk (Fig. 14c), could have originated if a very thin band, akin to the silvery striae in Tortricidae, appeared alongside the central symmetry system but later became decoupled from it and moved toward the apex of the wing. But again, “dBC” is considered to simply represent the distal margin of the central symmetry system.

The resemblance of certain *Sabatinca* wing patterns to the nymphalid groundplan suggests a revised version of Lemche’s “split-band” hypothesis for the origin of symmetry systems, in which symmetry systems originate from one-color bands that are bisected by another color and become concentric – but the location of these bands is constrained by veins at the costa, as postulated by the “wing-margin” model, instead of the points where veins bifurcate. This novel combination of two compatible concepts that had previously been discussed in isolation – the “wing-margin” model for band location and the “split-band” hypothesis for the origin of symmetry systems – fits the nymphalid groundplan very closely. Because the wing patterns of *Sabatinca doroxena* and *S. aurella* so closely match the nymphalid groundplan, the revised hypothesis presented here is currently better supported by empirical data than Lemche’s original “split-band” hypothesis and Nijhout's “fused-spot” hypothesis that had been proposed previously.

### Comparison with developmental mechanisms known from other Lepidoptera

The earliest studies of wing pattern evolution in Lepidoptera were based primarily on morphology, with preliminary phylogenetic context [47–49]. The first rigorous examination of wing pattern morphology in the context of phylogeny is over a century old [50] and is roughly contemporaneous with influential studies of other aspects of lepidopteran morphology [51]. In the wake of the publication of the nymphalid groundplan [24, 25], comparative morphology was a popular area of study that overlapped heavily with early experimental work on heredity [21, 22, 52–54]. During the current era, morphological insights continue to inform our understanding of the systematics of Lepidoptera [55, 56] and of the nymphalid groundplan [26]. Wing pattern homologies are designated through a combination of developmental, phylogenetic, and morphological data [57], and morphological data continue to shed light on the developmental genetics of color pattern, particularly when combined with phylogeny [58]. Developmental studies of wing pattern in Micropterigidae are not possible at present because no lab colony has been established, despite years of effort. However, current knowledge of the genetic underpinnings of wing pattern in macrolepidoptera and other panorpoid insects includes developmental mechanisms that may be relevant to Micropterigidae.

Expression of the developmental morphogen *wingless* precedes the development of wing pattern elements in many families of Lepidoptera [59], and also precedes the development of spots that are associated with venation in certain species of *Drosophila* [60]. In various macrolepidoptera, *wingless* is implicated in the development of two elements of the nymphalid groundplan [46, 59]: first, the discal spot (“Discalis I”) a wing pattern element that terminates near the costal margin of the wing and is associated with vein forks, but which does not correspond to any wing pattern element in Micropterigidae (Fig. 14); and second, the basal symmetry system (sensu Otaki [26]), also called “Discalis II,” which corresponds with the pattern element that terminates along the costal margin basal to Sc_1_ in *Sabatinca doroxena* and *S. aurella*.

The gene product *WntA* is involved with the development of various symmetry systems on butterfly wings, and is sufficiently well understood that its expression can be used to identify pattern homologies in situations where morphology is confusing or unclear [46]. In theory, the wing patterns of alternating light and dark stripes seen in Micropterigidae could develop as a result of the expression of *wingless* and *WntA*. Certain existing studies that use the nymphalid groundplan as a starting point have included various lineages of moths [59]; the results presented here can be used to guide the identification of nymphalid groundplan pattern elements in microlepidoptera, facilitating further taxonomic expansion of developmental studies.

### Ancestral states and the nature of wing pattern homology

A comparison of wing pattern in *Micropterix* and *Sabatinca* shows that the color of each pattern element can confound identification of homologies. The contrast boundaries that divide pattern elements, as opposed to the colors of pattern elements themselves, are the best indicators of homology.

**Fig. 15:**
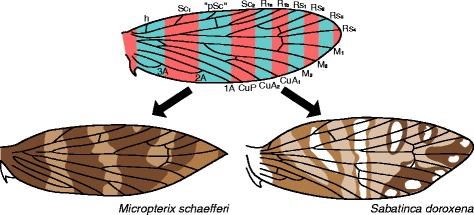
A new concept for wing pattern homologies. Micropterigid wing patterns are comprised of two series of alternating bands. Homologies can be recognized based on the location of contrast boundaries; pattern element color is not a reliable indicator of evolutionary history

Because the basal *Sabatinca* species with the most obviously fasciate wing patterns – *Sabatinca doroxena* and *S. aurella* (Fig. 5d, e) – have alternating light and dark bands straddling or abutting one vein each along the costa, just as in *Micropterix*, it is extremely likely that the wing pattern elements of *Sabatinca* and *Micropterix* are homologous. Even the shapes of individual wing pattern elements are similar between *Sabatinca* and *Micropterix*: for example, in *S. doroxena* and *S. aurella*, the band that straddles R_1a_ is quite small and does not even extend halfway toward the dorsum from the costa; the same is true for the wing pattern element that corresponds with R_1a_ in *Micropterix* [12]. However, the pattern element that straddles R_1a_ is of a light color on the wing of *Micropterix* (Fig. 1) and would therefore be called an “interfascial area” according to the “wing-margin” model [13, 14], but is surrounded by dark scales in *Sabatinca* and would therefore be called a “fascia” according to the same model. This, along with a similar observation from the “southern sabatincoid” genera discussed above and below, suggests that lepidopteran homologies between fasciae and interfascial areas, should they exist, occur among contrast boundaries as opposed to wing pattern element color. One contrast boundary occurs between each pair of adjacent plesiomorphic veins, and a series of alternating light and dark transverse bands will develop such that one band occurs within each pair of adjacent contrast boundaries. Either series of alternating bands – the series that straddles [h, “pSc”, R_1a_, Rs_1_, Rs_3_] or the series that straddles [Sc_1_, Sc_2_, R_1b_, Rs_2_, Rs_4_] – could develop a darker scale color (Fig. 15). In other words, the color of wing pattern elements is likely a misleading signifier of homology, with the contrast boundaries between pattern elements being far more reliable. Terms such as “ground color” and “background color” may be convenient for use in taxonomic descriptions but appear not to be meaningful in the context of evolutionary history.

**Fig. 16:**
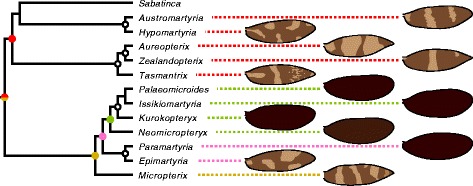
Wing pattern of Micropterigidae other than *Sabatinca* in the context of the preliminary phylogeny published by Gibbs & Lees [39]. Colored dots indicate nodes with over 90 % bootstrap support that include four or more species; corresponding colors are used to connect each species’ name with its illustrated wing pattern. Black-and-white dots indicate nodes with over 90 % bootstrap support that include two or three species

Reconstruction of an ancestral wing pattern state for all Micropterigidae is problematic at present because the rate of discovery of Gondwanan taxa remains high, and so various genera are not represented in the current, preliminary phylogeny. However, present knowledge supports some tentative conclusions. Firstly, it appears that bands are far more likely than spots to the primitive wing pattern element for Micropterigidae. The most basal Laurasian genus, *Micropterix*, has a wing pattern comprised entirely of bands. In the Gondwanan clade, bands predominate over spots in *Sabatinca* and are even more overwhelmingly dominant in all other genera: spots appear only in *Tasmantrix*, and occur far less consistently between species than bands in this genus (Fig. 12). The predominance of bands in the most basal Laurasian genus, and in all Gondwanan genera that could be described as “basal” Micropterigidae (Fig. 16), strongly indicates that ancestral Micropterigidae had banded wing patterns. Secondly, because both the Laurasian and Gondwanan clades include taxa with alternating dark and light bands straddling veins along the costal margin, the common ancestor of Micropterigidae most likely had the potential to develop a wing pattern similar to that of *Micropterix* that could later become confluent in many areas, leading to a wing pattern with fewer apparent wing pattern elements. Third, because the basal genera *Micropterix* and *Tasmantrix* both have a light band along the “pSc” region of the costa, surrounded on either side by dark bands that straddle Sc_1_ and Sc_2_, it appears most likely that ancestral Micropterigidae had dark bands straddling the veins [Sc_1_, Sc_2_, R_1b_, Rs_2_, Rs_4_], with the sabatincoid groundplan of dark bands straddling [h, “pSc”, R_1a_, Rs_1_, Rs_3_] originating later (Fig. 16).

### The number of primitive fasciae in Lepidoptera

Our results suggest that the primitive number of fasciae in Lepidoptera is less than seven. Wing patterns with seven or more fasciae have likely originated convergently in many lineages, perhaps evolving from the spots found in certain derived species of *Sabatinca*.

Though the “wing-margin” and “vein-fork” models share the assumption that transverse bands are the primitive wing pattern element for Lepidoptera, the two models differ in that the “vein-fork” model proposed a primitive groundplan with seven dark bands whereas the “wing-margin” proposes a primitive groundplan with either five or six dark bands, depending on whether Rs_4_ terminates along the costa. *Sabatinca doroxena* has a wing pattern of five dark bands that is entirely consistent with the “wing-margin” model’s prediction about the location of contrast boundaries (Fig. 5d). The band that straddles the humeral vein is part of a V-shaped pattern element that could have originated from a fasciate wing pattern in one of two ways: either two dark bands became confluent at the dorsal margin, or one dark band was split by a light pattern element that runs from the costa nearly to the dorsum. In *Sabatinca aurella*, this putative split is complete: the two apparent dark bands appear basal to Sc_1_ along the costa, with one straddling the humeral vein and one that does not straddle any vein (Fig. 5e). There are two simple explanations for this apparent split from one dark band into two. The first is that only one dark band occurred basal to Sc_1_ in ancestral *Sabatinca*, with this band nearly split in *S. doroxena*, and apparently completely split into two bands in *S. aurella* – though these features in both species are derived from a single primitive band. The second explanation is that two dark bands occurred basal to Sc_1_ in ancestral *Sabatinca*, which would require an additional two plesiomorphic branches of Sc – completely unknown from Trichoptera as well as Lepidoptera – to influence the development of wing pattern in extant Lepidoptera. The first explanation, of one primitive band that appears to split into two, is far more conservative in that it does not require the presence of plesiomorphic veins unknown from crown Amphiesmenoptera (the clade that includes all living moths and caddisflies), and is arguably also the more plausible of the two explanations given that multiple bands preceding Sc_1_ are not known from any micropterigid genus besides *Sabatinca*.

**Fig. 17:**
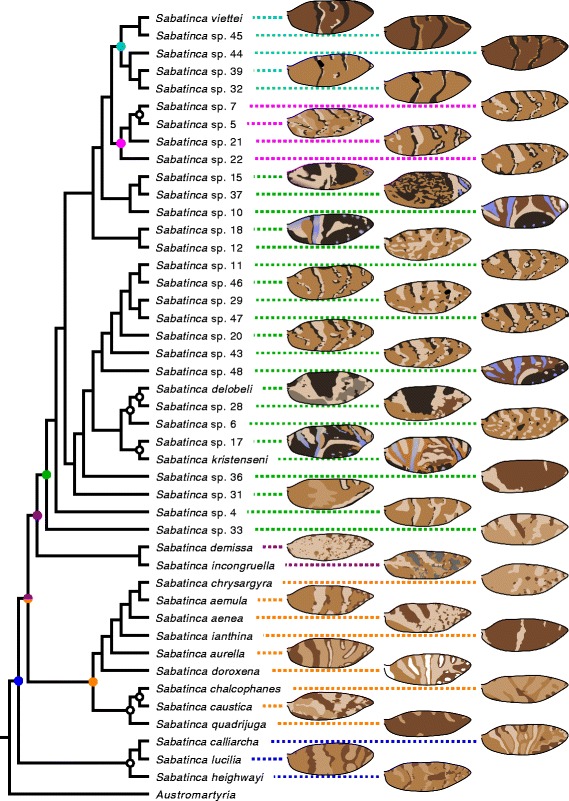
Wing pattern of *Sabatinca* in the context of the preliminary phylogeny published by Gibbs & Lees [39]. Colored dots indicate nodes with over 90 % bootstrap support that include four or more species; corresponding colors are used to connect each species’ name with its illustrated wing pattern. Black-and-white dots indicate nodes with over 90 % bootstrap support that include two or three species

**Fig. 18:**
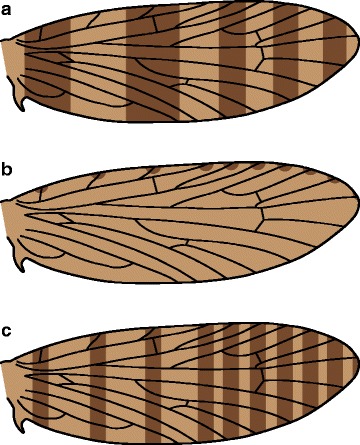
Possible origination of wing patterns with more than six fasciae. **a** Ancestral wing patterns are comprised of dark bands straddling alternating veins, as in *Micropterix*, *Sabatinca doroxena*, etc. **b** Derived wing patterns include spots that straddle all veins at the costa, as in *Sabatinca demissa*. **c** Each spot along the costa could extend into a band that reaches the dorsum

Even in the unlikely circumstance that two additional contrast boundaries between h and Sc_1_ are a reality, a groundplan with these additional contrast boundaries could only include the seven dark bands proposed by Lemche on wings where Rs_4_ terminates along the costa, and this rarely occurs. One possible groundplan that could underlie wing patterns with seven dark transverse bands would include individual dark bands straddling every vein at the costa, instead of straddling alternating veins. Dark spots such as those that have accumulated around each vein at the costa in various *Sabatinca* species (e.g., *S. caustica*, *chalcophanes*, *chrysargyra*, *demissa*, sp. 6, sp. 12) – many of which are distantly related (Fig. 17) – could extend down toward the dorsal margin of the wing in order to form bands (Fig. 18). This phenomenon would initially produce a groundplan with over seven bands, but the number of bands could be reduced to seven through confluence. *Sabatinca* sp. 48 (Fig. 8a) is the species that comes closest to exemplifying this sort of wing pattern, with dark scales straddling every vein at the costa and forming band-like elements, and light bands terminating between many adjacent vein pairs along the costa. This hypothesized, homoplastic, seven-band groundplan could be tested in future studies by examining the relationship between wing venation and color pattern in additional microlepidopteran lineages.

### Implications for Lemche’s “vein-fork” model

Though micropterigid wing patterns overwhelmingly do not conform to Lemche’s “vein-fork” model, a few species do offer insight into the processes that inspired the model.

Lemche’s “vein-fork” model for homology between wing pattern elements was originally based on observations of the location of spots on the wings of Pyralidae and Noctuidae [21]. Lemche found that spots often occurred at the points where veins bifurcate, and he extrapolated this observation from spots to fasciae, predicting that the basal edges of fasciae should also lie along points where veins bifurcate. This model therefore implies that fasciae and spots are homologous; because Lemche hypothesized that fasciae are the primitive wing pattern elements for Lepidoptera [22], one would expect that the spots in Pyralidae, Noctuidae, and many other moths to have arisen via incomplete expression of bands.

The “vein-fork” model initially appeared to be irrelevant to Micropterigidae, and therefore quite possibly of limited relevance to the evolution of wing pattern in Lepidoptera, because the model does not predict the location of fasciae in *Micropterix* [12]. The data presented here show that the model is of similarly limited utility for predicting the location of fasciae on the wings of other micropterigid taxa. However, in two *Sabatinca* species that appear to be distantly related – *S. demissa* (Fig. 6b) and *S*. sp. 6 (Fig. 7e) – prominent large, dark spots occur at many of the points where veins reach the wing margin and also at the point where the M vein bifurcates. In *Sabatinca demissa*, additional dark spots occur where Rs and CuA bifurcate; many more spots occur elsewhere on the wing, but are either much smaller or much lighter in color than those that occur where veins meet the wing margin and where M bifurcates. It is striking that the largest dark spots appear in the same locations relative to venation in both *Sabatinca* sp. 6 and *S. demissa*, because these two species’ color patterns are otherwise dissimilar: *S*. sp. 6 has large spots and bands that are very light in color whereas *S. demissa* has small, medium-brown spots against a very light ground color. Because prominent spots at the bifurcation of M are rare in Micropterigidae and have likely originated independently twice in *Sabatinca* alone (Fig. 17), it appears that Lemche erred in assuming that the spots in Pyralidae and Noctuidae are homologous and ancestral within and beyond the Lepidoptera [21]. The origination of such spots at vein bifurcations may well be a real phenomenon, but appears to have occurred convergently in various lepidopteran lineages and would therefore be homoplastic.

### Wing patterns with two colors

Both models put forth to explain wing pattern homology in microlepidoptera – the “wing-margin” model and the “vein-fork” model – assume a wing pattern that, at a first approximation, is comprised of one relatively light color and one relatively dark color. Though both models are based on taxa whose wing patterns include more than two colors, evaluation of these models is most straightforward for taxa whose wing patterns include only two colors. Six of the genera examined here – *Austromartyria*, *Hypomartyria*, *Aureopterix*, *Zealandopterix*, *Tasmantrix*, and *Epimartyria* – have wing patterns with only one light and one dark shade of brown, and all of these wing patterns are consistent with the “wing-margin” model. However, none of these genera provide as robust a test of the model as *Micropterix* because none have more than four pairs of alternating light and dark bands.

Other than *Micropterix*, *Epimartyria* (Fig. 13) is the only genus in the Laurasian, Northern Hemisphere clade whose wing pattern includes more than one color (Fig. 16). The wing patterns of both *E. bimaculella* and *E. pardella* could be said to be consistent with the “wing-margin” model, in that the single light pattern element at the costa straddles only one vein. However, because so few differentiated pattern elements occur on the wing of *Epimartyria*, this genus does not provide any additional insight into the applicability of the “wing-margin” model to Micropterigidae.

The wing patterns of all taxa in the “Australian group” are consistent with the “wing-margin” model as observed in *Micropterix*. The only light band that reaches the costa in most *Tasmantrix* species (Fig. 12) corresponds to the interfascial area that separates the subbasal and median fasciae in *Micropterix* (Fig. 1), and the light band that reaches the costa in *T. lunaris* and *T. tasmaniensis* corresponds to the interfascial area between the median and postmedian fasciae in *Micropterix*, with additional lack of expression of the postmedian fascia at R_1b_ in *T. tasmaniensis*. The wing pattern of *Zealandopterix* (Fig. 11i) is dominated by dark pattern elements and consists of a small spot at the base of the wing that does not reach the costa, a smaller spot at the wing apex that does not straddle any veins, a light band that reaches the “pSc” area of the costa that separates the subbasal and median fasciae as in *Tasmantrix* (Fig. 12) and *Micropterix* (Fig. 1), and a light band that straddles Rs_1_, corresponding to the interfascial area that separates the postmedian and preterminal fasciae in *Micropterix*. Wing pattern in *Tasmantrix* and *Zealandopterix* is therefore consistent with the “wing-margin” model with confluence of fasciae due to suffusion of interfascial areas, plus with the addition of small light spots between fasciae. In contrast to the other genera in the “Australian group,” *Aureopterix* (Fig. 11g, h) has a wing pattern dominated by light pattern elements. Nevertheless, the wing pattern of *Aureopterix micans* (Fig. 11g) is broadly consistent with the *Micropterix* groundplan (Fig. 1): dark bands straddle Sc_1_ and Sc_2_ exactly as predicted by the “wing-margin” model, corresponding to the subbasal and median fasciae; R_1b_ is sometimes straddled by a dark band, corresponding to the postmedian fascia; and Rs_2_ is straddled by a dark band corresponding to the preterminal fascia, which has become confluent with the postmedian fascia. The wing pattern of *Aureopterix micans* can therefore be derived from the “wing-margin” model through lack of expression of the basal fascia and confluence of the postmedian and preterminal fasciae. The wing pattern of *Aureopterix sterops* (Fig. 11h) is very similar, except that the basal fascia is partially expressed, the subbasal fascia does not straddle Sc_1_ at the costa due to incomplete lack of expression, and the postmedian and preterminal fasciae are not confluent as frequently. Between *Aureopterix micans* and *A. sterops*, all fasciae and interfascial areas predicted by the “wing-margin” model are present with the sole exception of the terminal fascia, which is absent by necessity because Rs_4_ does not terminate along the costa in *Aureopterix*.

In the two “southern sabatincoid” genera whose wing patterns contain two colors, *Austromartyria* and *Hypomartyria* (Fig. 11a, b), the only veins ever straddled by light bands are Sc_1_, Sc_2_, and R_1b_. These three veins form an alternating series, as they are interspersed between h, “pSc,” R_1a_, and Rs_1_, and so at first glance the southern sabatincoid wing patterns appear to be consistent with the “wing-margin” model. However, the model predicts that these veins should be straddled by dark, not light, bands (Fig. 1). The contrast boundaries between wing pattern elements in *Austromartyria* and *Hypomartyria* are consistent with those predicted by the “wing-margin” model, but the colors of these pattern elements are not – just as in *Sabatinca doroxena* and *S. aurella* (Fig. 15). Because *Austromartyria*, *Hypomartyria*, and *Sabatinca* form a single clade (Fig. 16), it appears that the common ancestor of all Micropterigidae had a wing pattern that conforms to the “wing-margin” model (Fig. 1) in terms of both contrast boundary location and pattern element color – as seen in *Micropterix*, *Tasmantrix*, *Zealandopterix*, and *Aureopterix* – and that the common ancestor of *Austromartyria*, *Hypomartyria*, and *Sabatinca* had a wing pattern in which the dark pattern element of *Micropterix* became light, and vice-versa.

Only three of the *Sabatinca* species examined have wing patterns with just two colors: *S. quadrijuga* (Fig. 5a), *S. ianthina* (Fig. 5f), and *S.* sp. 36 (Fig. 7b). The only light wing pattern elements that reach the costa in *Sabatinca quadrijuga* straddle Sc_1_ and Sc_2_, just as in *Austromartyria* (Fig. 11a); no light wing patterns straddle any veins along the costa of *S. ianthina* or *S.* sp. 36. The latter two species, though phylogenetically and geographically distant from each other (Fig. 17), have very similar wing patterns: an overwhelmingly dark wing with very light pattern elements occurring basal to the humeral vein, in the “pSc” area, between R_1a_ and R_1b_, straddling or very close to Rs_4_, and between M_3_ and CuA_1_; *S. ianthina* also has a light band between Rs_2_ and Rs_3_ and small light spots between M_1_ and M_2_. This wing pattern could be derived from the *Micropterix* groundplan through complete suffusion of various interfascial areas, and incomplete suffusion of all others; both complete and incomplete suffusion of interfascial areas have been observed in various *Micropterix* species [12]. The small light bands along the costa in these two species correspond to the *Micropterix* interfascial area that straddles “pSc” and the *Micropterix* interfascial area that straddles R_1a_, with incomplete suffusion adjacent to the median fascia. The additional light band on the wing of *Sabatinca ianthina* corresponds to the interfascial area that straddles Rs_3_ in *Micropterix* (Fig. 1), with incomplete suffusion adjacent to the terminal fascia. The light band that straddles Rs_4_ in many *Sabatinca* sp. 36 specimens could be attributed to lack of expression of the terminal fascia along the dorsum and incomplete suffusion of the adjacent interfascial area along the costa. Both of these groundplan modifications have been observed in *M. rothenbachii*, though not in the same specimen [12]. Because few *Sabatinca* species have wing patterns comprised of only two colors, and because these wing patterns are characterized by extensive suffusion of interfascial areas, this genus adds little to our understanding of micropterigid wing patterns that are comprised strictly of one light shade and one dark shade of brown.

### Wing patterns with three or more colors

Wing patterns include three or more colors in *Nannopterix*, *Agrionympha*, and the vast majority of *Sabatinca* species. In *Nannopterix choreutes* (Fig. 11f), light scales straddle all veins at the costa except for Rs_2_ and so no firm conclusions can be drawn regarding the “wing-margin” model, or homology in any other sense. In *Agrionympha* (Fig. 11c-e), light bands are bordered by very dark, thin bands. Among various other possible mechanisms, these very dark bands could have arisen in the manner predicted by Lemche’s “split-band” hypothesis, with each pair of dark bands originating from a single, ancestral dark band that was bisected by a very light band. In the three *Agrionympha* species examined here, a light band and the two very dark bands that border it all fall within the “pSc” region, occasionally abutting Sc_1_ but never straddling either of the Sc veins expressed in the adult wing; because all two-colored micropterigid wing patterns are consistent with the “wing-margin” model, which predicts a single wing pattern element in the “pSc” are between Sc_1_ and Sc_2_, this suggests that each light band, plus the two very dark bands that border it, function together as a single wing pattern element. The three *Agrionympha* species examined have a another “split-band”-type wing pattern element at R_1_, but because this pattern element straddles different veins in different taxa – R_1b_ in *A. capensis* and *A. fuscoapicella* and R_1a_ in *A. sagittella* – it is difficult to determine how this pattern element, and therefore *Agrionympha* wing patterns as a whole, might relate to the “wing-margin” model.

In *Sabatinca*, the relationships between wing pattern elements of different colors seem to vary greatly among species. For example, in the *chrysargyra* group (Fig. 5) – a small, well-supported clade – *Sabatinca doroxena* and *S. aurella* have fasciate wing patterns in which the one or two most basal dark bands are of a single color, but all others are bisected by a very light color (Fig. 5d, e). These wing patterns essentially provide an illustration of the “split-band” hypothesis, because the basal bands conform exactly to Lemche’s hypothesized ancestral state for microlepidopteran wing pattern and the others conform exactly to Lemche’s hypothesized incipient symmetry systems. A few other *Sabatinca* species, such as *S. lucilia* (Fig. 4c), have wing pattern elements that somewhat resemble the “split-band,” but not as unambiguously so. In *Sabatinca caustica* and *S. chalcophanes* (Fig. 5b, c), the darkest wing pattern elements occur only at the costal and dorsal wing margins and are connected by medium-brown bands. In *Sabatinca chrysargyra* (Fig. 5i), the darkest pattern elements are small spots that straddle veins at the wing margin and the lightest pattern elements are much larger bands that do not straddle veins.

In the *incongruella* group, three-color wing patterns are even more varied. *Sabatinca demissa* (Fig. 6b) has large, dark spots at the points where veins reach the costa or bifurcate, and small, light spots elsewhere. In *Sabatinca* sp. 33 (Fig. 6c), only the lightest and darkest wing pattern elements reach the costa, with the exception of Rs_3_ in some specimens. *Sabatinca* sp. 6 (Fig. 7e) and the distantly related *S*. sp. 12 (Fig. 9a) have color patterns very similar to that of *S. chrysargyra* (Fig. 5i). Many *Sabatinca* species from New Caledonia have wing patterns somewhat similar to the “split-band”-type patterns of *Sabatinca doroxena* and *S. aurella* from New Zealand, but the thin, dark bands of *S. doroxena* and *S. aurella* often do not appear as bands at all in the New Caledonian species and instead are either broken up into small spots or are absent altogether, particularly at the apical, or distal, margin of each light band. However, the relationship between patterning and venation differs markedly between the “split-band”-type *Sabatinca* species of New Zealand and New Caledonia: whereas the “split-band”-type pattern elements straddle alternating veins along the costa in the New Zealand species (Fig. 5d, e) and in *Sabatinca* sp. 31 from New Caledonia (Fig. 7a), it is common for every single vein along the costa to be surrounded or abutted by a “split-band”-type pattern element in New Caledonian species (Figs. 8b-g and 10b, h).

*Sabatinca* sp. 37 (Fig. 9e) has a wing pattern of only two colors except at the apical area. Its wing pattern is not exactly fasciate – if this wing pattern is indeed derived from an ancestral fasciate pattern, the edges of the fasciae have become rather sinusoidal, creating a reticulate pattern comprised of elements that simultaneously resemble both fasciae and spots. However, these sinusoidal fasciae do straddle alternating veins along the costa: the area basal to Sc_1_, the “pSc” area, R_1a_, and Rs_1_. (Rs_3_ is straddled by a light band that also straddles both Rs_2_ and Rs_4_.) Because the various colors on the wing of *Sabatinca* sp. 37 are limited to constrained to small portions of the wing, potential relevance to the “wing-margin” model may be easier to deduce.

**Fig. 19:**
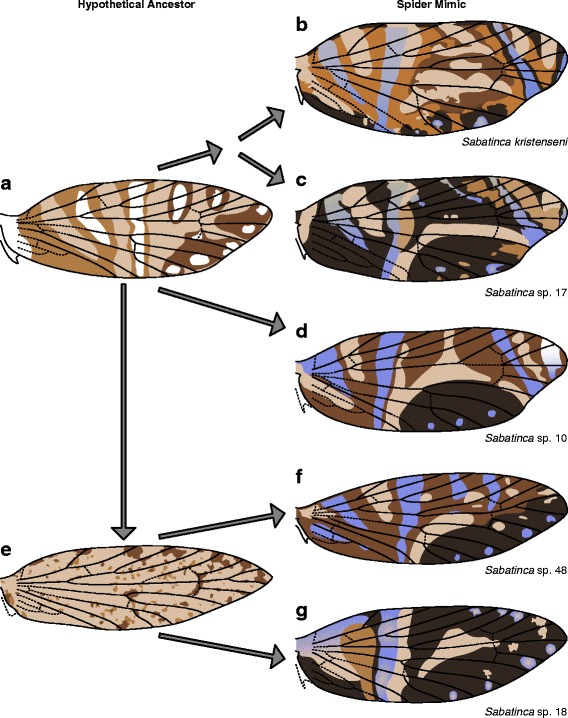
Various ways to construct Possible origins for various spider-mimic wing patterns. **a** An ancestral wing pattern with fasciae straddling alternating veins at the costa, as seen in *Sabatinca doroxena*. **b**, **c**, **d** “Spider-mimic” wing patterns in which the vestiges of such a fasciate pattern can still be seen. **e** A derived wing pattern with dark spots straddling all veins at the costa, as seen in *Sabatinca demissa*. **f**, **g** “Spider-mimic” wing patterns in which all veins are surrounded by dark scales at the costa

Many *Sabatinca* species from New Caledonia have wing patterns with spots and transverse bands in light brown, dark brown, and iridescent blue; these wing patterns have been hypothesized to mimic the jumping spiders that appear on the island [39]. These spider-mimic patterns appear to have evolved convergently in multiple lineages (Fig. 17), and indeed, wing patterns in distinct spider-mimic clades appear to be constructed differently (Fig. 19). Dark scales surround (or in one case, abut) every single vein at the costa in *Sabatinca* spp. 48 and 18 (Figs. 8a and 9b). This sort of wing pattern may be derived from ancestral pattern in which dark spots appeared around all veins along the costa. In *Sabatinca kristenseni*, *S*. sp. 17, and *S*. sp. 10 (Figs. 7c-d and 9c), vestiges of the “wing-margin” model are easily observed. In the sister species *Sabatinca kristenseni* and *S*. sp. 17, Sc_2_, Rs_2_, and Rs_4_ are surrounded by light brown or light blue bands and Rs_1_ is surrounded by a dark band. R_1a_ is surrounded by dark scales in *S. kristenseni* and abutted by dark scales in *S*. sp. 17; Sc_1_, R_1b_, and Rs_3_ are abutted by light and dark pattern elements in both species. The wing patterns of *Sabatinca kristenseni* and *S*. sp. 17 therefore retain a shared resemblance to the wing patterns of *S. doroxena* and *S. aurella*. The same is true for *S*. sp. 10, although different veins are surrounded entirely by light scales: Sc_1_ and R_1b_ – just as in *S. doroxena* and *S. aurella* – and also Rs_3_, which is surrounded by a band in a different, whitish color.

In summary, third and fourth colors in *Sabatinca* wing patterns seem to have originated independently multiple times and through a variety of mechanisms, often obscuring homologies with more straightforward pattern elements seen on the wings of other micropterigid genera with wing patterns comprised of only two colors, such as *Tasmantrix*, *Austromartyria*, and *Micropterix*. In *Sabatinca* sp. 37 the four colors in the wing pattern are largely confined to specific areas along the proximo-distal axis and in *S.* sp. 33 colors are confined to specific areas along the anterior-posterior axis, but in all other species, different colors are dispersed throughout the wing. In *Sabatinca demissa*, the color of a spot corresponds with its proximity to the points where veins bifurcate and terminate. In *Sabatinca* sp. 6, the darkest spots along the costal margin always straddle veins and the lightest spots never do. In *Sabatinca doroxena* and *S. aurella*, the lightest color on a wing, either beige or white, seems to have originated within the central areas of the dark brown bands. In various putative spider mimics, blue and light brown bands are adjacent to each other and may have originated when one band split into two, losing its self-symmetry. Many species from New Caledonia have transverse light bands surrounded by dark bands or spots on both sides; there is a consistent relationship between venation and both of the band colors – light bands always straddle Sc_1_ and Sc_2_, and a dark band always straddles R_1a_ – suggesting that both of these colors, whether they arose from the hypertrophy seen in *Sabatinca doroxena* and *S. aurella* or by some other mechanism, are developmentally individuated.

## Conclusions

From an examination of micropterigid wing patterns that are comprised of two colors, it appears that the ancestral state for this family – and therefore quite possibly for the order Lepidoptera – is a wing pattern of alternating light and dark bands, with each band straddling one vein along the costa. This ancestral state conforms to the predictions of the “wing-margin” model, originally based on Tortricidae [13, 14]. However, a comparison of the wing patterns of *Micropterix* with *Sabatinca doroxena* and *S. aurella* shows that the “wing-margin” correctly predicts the location of transverse bands and the contrast boundaries between them, but cannot predict which series of bands will be light brown and which will be dark brown. The wing pattern elements of *Sabatinca doroxena* and *S. aurella* – simple bands of a single dark color, and two-color bands in which dark scales surround a central light area – illustrate both stages of “split-band” symmetry system formation hypothesized by Lemche [22], thus strongly supporting his hypothesis that symmetry systems originated when dark bands were bisected, or hypertrophied, by light bands. When the wing pattern of *Sabatinca doroxena* is plotted on to a nymphalid wing following the constraints proposed by the “wing-margin” model, the resulting hypothetical wing pattern very strongly resembles the nymphalid groundplan. Because the “wing-margin” model correctly predicts the location of wing pattern elements in distantly related lepidopteran lineages (Micropterigidae, Tortricidae), and, in combination with the “split-band” hypothesis, can predict the nymphalid groundplan based on wing pattern in *Sabatinca*, the “wing-margin” model and the “split-band” hypothesis appear to have great potential to explain wing pattern diversity in the order Lepidoptera.

## Methods

The specimens examined for this study are held in the Australian National Insect Collection in Canberra, Australia; Victoria University in Wellington, New Zealand; and the Smithsonian Institution in Washington DC, USA. Only forewings were examined, because hindwings have very light scales of only one color. A total of 918 wings were examined, which may have included one or both forewings from a given specimen – the only wings that were excluded are those in which the relationship between venation and patterning cannot be deduced because the scales are worn off or the wing is broken. These 918 wings represent 66 species and 9 genera of Micropterigidae. Taxa were selected to match those sampled in the existing preliminary micropterigid phylogeny [39]. Sampling differences between the preliminary phylogeny and the present study are as follows: *Micropterix* was not included here because this genus has already been examined [12]; *Sabatinca* spp. 5b, 49, and 50 were not included here because these species are only known from specimens preserved in ethanol; and *Epimartyria auricrinella* and the genera *Paramartyria*, *Palaeomicroides*, *Issikiomartyria*, *Kurokopteryx*, and *Neomicropteryx* were not included here because these species’ wings are of only a single color, a dark brown similar to the color of dark bands in *Micropterix* [20, 40]. Additional species belonging to the genera *Epimartyria* (*E. bimaculella*) and *Tasmantrix* (*T. calliplaca, T. lunaris, T. nigrocornis, T. phalaros,* and *T. tasmaniensis*), and species representing the additional genera *Agrionympha* (*A. capensis, A. fuscoapicella,* and *A. sagittella*) and *Nannopterix* (*N. choreutes*) were included here, despite being absent from the preliminary phylogeny, because specimens were available and because the affinities of these additional genera have already been discussed in the literature [42, 61].

The methods used here to examine wing pattern morphology parallel those developed by Schachat and Brown [12] and are as follows: For each species, one forewing from one specimen was selected to form the basis of the illustration of that species’ wing pattern. The wings selected were those that had intact color pattern, minimal overlap between the forewing and hindwing, and minimal overlap between the wing and the small block holding the minuten pin. This allowed maximum light to shine through the backlit wing. Scaled wings, instead of cleared wings, were examined in order to observe the precise relationship between color pattern and venation. Micropterigid wings are thinly scaled, and the venation becomes visible when specimens are lit from below using a microscope stage light. The observed wing venation was confirmed by examination of published illustrations of wing venation [40, 61, 62] and by examination of a wing slide prepared by Don Davis and Jean-Francois Landry and held at the USNM for *Epimartyria bimaculella*, and by examination of wing slides prepared by George Gibbs and held at Victoria University for all other species; for the 7 species for which wing slides are not available (*Agrionympha capensis*, *A. sagittella*, *A. fuscoapicella*, *Sabatinca viettei*, and *S*. spp. 36, 39, and 43;), the wing slide of a sister species was examined for *Sabatinca* and published illustrations were consulted for *Agrionympha* [61].

To verify that the illustrations fully represent the species to which they correspond, a total of up to 20 forewings were examined under a light stereomicroscope. (Results are discussed primarily in terms of wings instead of specimens because, in a few cases, only one forewing could be examined per specimen due to wear, due to the angle at which the specimen had been pinned, or because one wing had been removed to make a wing slide. Furthermore, a number of specimens have color patterns that varied between the two forewings.) Variations were noted at all locations along the costa where veins terminate, with the frequent exception of the humeral vein, which often cannot be detected on scaled specimens. Variations were also noted in between the two visible branches of the Sc vein, because an ancestral vein in this location has been hypothesized to constrain wing pattern [12], and variations were noted at the location where the Rs_4_ vein terminates because, although this vein does not terminate along the costa in any of the species examined for the present study, it does terminate along the costa in *Micropterix* [43] and occasionally in fossil Micropterigidae [63]. To create illustrations, a forewing was photographed while backlit so that both the patterning and venation were visible. This photograph was used as a template for a wing venation/wing patterning illustration created in the vector graphics application Affinity Designer. All intraspecific variation was incorporated into one single illustration per species. For each species, the illustrated wing pattern is that which is most prevalent; each variation is noted by a number and illustrated with a line comprised of red dashes alternating with the color that is present in the variation. Furthermore, supplemental material includes a written description of each pattern variation as well as prevalence data (Additional file 1: Tables S1, S2, S3).

The location of the wing vein 1A + 2A could not be observed in all pinned specimens because of the overlap between the forewing and hindwing, and therefore had to be inferred based on wing slides and previously described venation [40, 61, 62]; however, this vein is of no relevance to the model because it does not reach the costal margin. Similarly, the jugal lobe was often folded in the specimens examined; its outline was inferred based on wing slides and previous descriptions. These and other inferred features are illustrated with dashed lines. In descriptions presented in the Results, the humeral vein is often excluded from statements regarding wing veins that terminate along the costa, because this vein is so often difficult to observe.

## Additional file

Additional file 1: Table S1. Non-*Sabatinca* wing pattern variation. **Table S2.** *Sabatinca* wing pattern variation from New Zealand. **Table S3.** *Sabatinca* wing pattern variation from New Caledonia. (DOC 145 kb)
