# Supplementary material for: Forewing color pattern in Micropterigidae (Insecta: Lepidoptera): homologies between contrast boundaries, and a revised hypothesis for the origin of symmetry systems
Source: BMC Evol Biol. 2016 May 26;16:116. doi: 10.1186/s12862-016-0687-z (PMC4880886; doi:10.1186/s12862-016-0687-z)
Supplement: Additional file 1: Table S1. — Non-Sabatinca wing pattern variation. Table S2. Sabatinca wing pattern variation from New Zealand. Table S3. Sabatinca wing pattern variation from New Caledonia. (DOC 145 kb) [file 12862_2016_687_MOESM1_ESM.doc]

Additional file 1

**Table S1**. Non-*Sabatinca* wing pattern variation

| **Collection** | **Genus** | **Species** | **GP** | **V1** | **V2** | **V1&2** |
| --- | --- | --- | --- | --- | --- | --- |
| ANIC | *Austromartyria* | *porphyrodes* | 14 | 2 | 4 |  |
| VUW | *Hypomartyria* | *micropteroides* | 1 |  |  |  |
| VUW | *Agrionympha* | *capensis* | 19 | 1 |  |  |
| VUW | *Agrionympha* | *sagitella* | 19 | 1 |  |  |
| VUW | *Agrionympha* | *fuscoapicella* | 6 |  |  |  |
| VUW | *Aureopterix* | *micans* | 16 | 2 | 2 |  |
| ANIC | *Aureopterix* | *sterops* | 9 |  | 8 | 3 |
| VUW | *Nannopterix* | *choreutes* | 12 |  |  |  |
| VUW | *Zealandopterix* | *zonodoxa* | 8 |  | 8 | 4 |
| ANIC | *Tasmantrix* | *calliplaca* | 20 |  |  |  |
| ANIC | *Tasmantrix* | *fragilis* | 12 |  |  |  |
| ANIC | *Tasmantrix* | *lunaris* | 8 | 2 |  |  |
| ANIC | *Tasmantrix* | *nigrocornis* | 18 | 2 |  |  |
| ANIC | *Tasmantrix* | *phalaros* | 16 | 4 |  |  |
| ANIC | *Tasmantrix* | *tasmaniensis* | 10 | 1 | 3 |  |
| ANIC | *Tasmantrix* | *thula* | 18 | 2 |  |  |
| USNM | *Epimartyria* | *bimaculella* | 8 | 2 | 7 | 3 |
| ANIC, USNM | *Epimartyria* | *pardella* | 10 | 7 | 1 |  |

**Legend:** ANIC: Australian National Insect Collection, VUW: Victoria University Wellington, USNM: United States National Museum, GP: Groundplan (as illustrated), V1 = Variation 1, etc.

Explanation of variations:

- *Austromartyria porphyrodes*

1. Sc_1_ straddled by light scales at costa
2. Sc_2_ surrounded by light scales at costa

- *Agrionympha capensis*

1. R_1a_ surrounded by darkest scales at costa

- *Agrionympha sagitella*

1. R_1a_ surrounded by darkest scales at costa

- *Aureopterix micans*

1. R_1b_ surrounded by dark scales at costa
2. Rs_2_ surrounded by light scales at costa

- *Aureopterix sterops*

1. R_1b_ surrounded by dark scales at costa
2. Rs_1_ surrounded by dark scales at costa

- *Zealandopterix zonodoxa*

1. Light area between Sc_1_ and Sc_2_ does not reach the costa
2. Rs_1_ surrounded by dark scales at costa

- *Tasmantrix lunaris*

1. R_1a_ surrounded by light scales at costa

- *Tasmantrix nigrocornis*

1. Sc_1_ surrounded by light scales at costa

- *Tasmantrix phalaros*

1. Sc_1_ surrounded by light scales at costa

- *Tasmantrix tasmaniensis*

1. Sc_1_ surrounded by light scales at costa
2. R_1a_ surrounded by dark scales at costa

- *Tasmantrix thula*

1. Sc_1_ surrounded by light scales at costa

- *Epimartyria bimaculella*

1. Sc_2_ surrounded by light scales at costa
2. R_1_ surrounded by dark scales at costa

- *Epimartyria pardella*

1. Sc_2_ surrounded by light scales at costa
2. R_1_ surrounded by dark scales at costa

**Table S2**. *Sabatinca* wing pattern variation from New Zealand

| **Species** | **GP** | **V1** | **V2** | **V3** | **V4** | **V 1,2** | **V 2,3** | **V 2,4** | **V 2,5** | **V 3,4** | **V 4,5** | **V1, 2,5** | **V5, 6,7** | **V2, 3,5, 6** | **V2, 3,5, 6,7** |
| --- | --- | --- | --- | --- | --- | --- | --- | --- | --- | --- | --- | --- | --- | --- | --- |
| *aemula* | 10 | 2 |  |  |  |  |  |  |  |  |  |  |  |  |  |
| *aenea* | 7 | 6 | 3 |  |  |  |  |  |  |  |  |  |  |  |  |
| *aurella* | 20 |  |  |  |  |  |  |  |  |  |  |  |  |  |  |
| *calliarcha* | 6 | 3 | 4 | 2 | 1 |  |  |  |  |  |  |  |  |  |  |
| *caustica* | 7 |  | 5 |  |  | 6 | 2 |  |  |  |  |  |  |  |  |
| *chalcophanes* | 7 |  | 4 |  |  |  | 1 | 2 | 2 |  | 2 | 2 |  |  |  |
| *chrysargyra* | 8 | 2 |  |  | 1 |  | 4 |  |  | 1 |  |  | 1 | 2 | 1 |
| *demissa* | 11 | 6 | 2 |  |  | 1 |  |  |  |  |  |  |  |  |  |
| *doroxena* | 8 | 2 | 2 |  |  |  |  |  |  |  |  |  |  |  |  |
| *heighwayi* | 5 | 1 |  |  |  |  |  |  |  |  |  |  |  |  |  |
| *ianthina* | 15 | 2 | 1 |  |  | 2 |  |  |  |  |  |  |  |  |  |
| *incongruella* | 1 |  |  |  |  |  |  |  |  |  |  |  |  |  |  |
| *lucilia* | 7 | 1 | 2 |  |  |  |  |  |  |  |  |  |  |  |  |
| *quadrijuga* | 12 | 2 |  |  |  |  |  |  |  |  |  |  |  |  |  |

All *Sabatinca* specimens examined are held in the collection of Victoria University, Wellington. **Legend:** GP: Groundplan (as illustrated), V1 = Variation 1, etc.

Explanation of variations:

- *Sabatinca aemula*

1. R_1b_ surrounded by light area at costa

- *Sabatinca aenea*

1. R_1b_ surrounded by dark area at costa
2. Rs_1_ surrounded by light area at costa

- *Sabatinca calliarcha*

1. Sc_1_ surrounded by light scales at costa
2. Sc_2_ surrounded by dark scales at costa
3. R_1a_ surrounded by light scales at costa
4. Rs_1_ surrounded by light scales at costa

- *Sabatinca caustica*

1. Sc_2_ surrounded by darkest scales at costa
2. R_1b_ surrounded by lightest scales
3. Rs_2_ surrounded by light scales at costa

- *Sabatinca chalcophanes*

1. h surrounded by dark scales at costa
2. Sc_2_ surrounded by dark scales at costa
3. R_1a_ surrounded by medium scales at costa
4. R_1b_ surrounded by dark scales at costa
5. Rs_2_ surrounded by dark scales at costa

- *Sabatinca chrysargyra*

1. Sc_1_ surrounded by darkest scales at costa
2. Sc_2_ surrounded by lightest scales at costa
3. R_1a_ surrounded by lightest scales at costa
4. R_1b_ surrounded by darkest scales at costa
5. Rs_1_ surrounded by lightest scales at costa
6. Rs_2_ surrounded by lightest scales at costa
7. Rs_3_ surrounded by lightest scales at costa

- *Sabatinca demissa*

1. R_1b_ surrounded by light scales at costa
2. Rs_2_ surrounded by light scales at costa

- *Sabatinca doroxena*

1. Sc_1_ surrounded by medium scales at costa
2. Sc_2_ surrounded by medium scales at costa

- *Sabatinca heighwayi*

1. Sc_2_ surrounded by medium brown scales at costa

- *Sabatinca ianthina*

1. Light area between R_1a_ and R_1b_ does not reach costa
2. Light area between Rs_2_ and Rs_3_ does not reach costa

- *Sabatinca lucilia*

1. R_1a_ surrounded by medium scales at costa
2. Rs_3_ surrounded by lightest scales at costa

- *Sabatinca quadrijuga*

1. Sc_2_ surrounded by dark scales at costa

**Table S3**. *Sabatinca* wing pattern variation from New Caledonia

| **Species** | **GP** | **V1** | **V2** | **V3** | **V4** | **V1,2** | **V2,3** | **V2,4** | **V3,4** | **V1,3,4** | **V2,3,4** |
| --- | --- | --- | --- | --- | --- | --- | --- | --- | --- | --- | --- |
| 4 | 10 | 2 | 1 |  |  | 7 |  |  |  |  |  |
| 5 | 6 |  |  |  |  |  |  |  |  |  |  |
| 6 | 11 | 2 | 7 |  |  |  |  |  |  |  |  |
| 7 | 10 | 2 | 8 |  |  |  |  |  |  |  |  |
| 10 | 18 | 2 |  |  |  |  |  |  |  |  |  |
| 11 | 18 | 2 |  |  |  |  |  |  |  |  |  |
| 12 | 9 | 6 | 4 |  |  |  | 1 |  |  |  |  |
| 15 | 8 |  |  |  |  |  |  |  |  |  |  |
| 17 | 12 |  |  |  |  |  |  |  |  |  |  |
| 18 | 10 | 2 |  |  |  |  |  |  |  |  |  |
| 20 | 9 | 5 | 4 |  |  | 2 |  |  |  |  |  |
| 21 | 6 | 4 |  |  | 2 |  |  |  | 2 | 1 | 1 |
| 22 | 17 |  | 1 |  |  | 1 | 1 |  |  |  |  |
| 28 | 11 |  |  |  |  |  |  |  |  |  |  |
| 29 | 6 |  |  | 6 |  | 4 |  |  |  |  |  |
| 31 | 2 |  |  |  |  |  |  |  |  |  |  |
| 32 | 12 |  |  |  |  |  |  |  |  |  |  |
| 33 | 4 | 1 | 2 |  |  |  |  |  |  |  |  |
| 36 | 6 | 4 | 2 |  |  |  |  |  |  |  |  |
| 37 | 2 |  |  |  |  |  |  |  |  |  |  |
| 39 | 1 |  |  |  |  |  |  |  |  |  |  |
| 43 | 2 |  |  |  |  |  |  |  |  |  |  |
| 44 | 13 |  |  |  |  |  |  |  |  |  |  |
| 45 | 5 | 4 |  |  |  |  |  |  |  |  |  |
| 46 | 7 | 1 | 1 | 1 | 4 |  |  | 2 | 2 |  |  |
| 47 | 7 | 1 |  |  |  |  |  |  |  |  |  |
| 48 | 12 |  |  |  |  |  |  |  |  |  |  |
| *delobeli* | 10 | 8 | 2 |  |  |  |  |  |  |  |  |
| *kristenseni* | 14 | 6 |  |  |  |  |  |  |  |  |  |
| *viettei* | 6 |  |  |  |  |  |  |  |  |  |  |

All *Sabatinca* specimens examined are held in the collection of Victoria University, Wellington. **Legend:** GP: Groundplan (as illustrated), V1 = Variation 1, etc.

Explanation of variations:

- *Sabatinca* sp. 4

1. Sc_1_ surrounded by dark scales at costa
2. Sc_2_ surrounded by dark scales at costa

- *Sabatinca* sp. 6

1. R_1a_ surrounded by medium scales at costa
2. R_1b_ surrounded by medium scales at costa

- *Sabatinca* sp. 7

1. Rs_1_ surrounded by medium scales at costa
2. Rs_3_ surrounded by darkest scales at costa

- *Sabatinca* sp. 10

1. R_1b_ surrounded by brown scales at costa

- *Sabatinca* sp. 11

1. R_1b_ surrounded by medium scales at costa

- *Sabatinca* sp. 12

1. Sc_1_ surrounded by medium scales at costa
2. R_1b_ surrounded by medium scales at costa
3. Rs_1_ surrounded by medium scales at costa

- *Sabatinca* sp. 18

1. Sc_1_ surrounded by bluish scales at costa

- *Sabatinca* sp. 20

1. R_1b_ surrounded by medium scales at costa
2. Rs_2_ surrounded by medium scales at costa

- *Sabatinca* sp. 21

1. The distal edge of R_1b_ is abutted by medium scales at the costa
2. Rs_1_ surrounded by medium scales at costa
3. Rs_2_ surrounded by medium scales at costa
4. Rs_3_ surrounded by medium scales at costa

- *Sabatinca* sp. 22

1. R_1a_ surrounded by medium scales at costa
2. R_1b_ surrounded by darkest scales at costa
3. Rs_2_ surrounded by darkest scales at costa

- *Sabatinca* sp. 29

1. R_1a_ surrounded by darkest scales at costa
2. Rs_1_ surrounded by darkest scales at costa
3. Rs_3_ surrounded by lightest scales at costa

- *Sabatinca* sp. 33

1. Darkest scales don't reach costa at area between h and Sc_1_
2. Darkest scales surround costa at Rs_3_

- *Sabatinca* sp. 36

1. Light scales meet costa between Sc_1_ and Sc_2_
2. Dark scales straddle/abut Rs_4_ at termen

- *Sabatinca* sp. 45

1. R_1b_ surrounded by medium scales at costa; the dark band that straddles Rs_1_,_2_,_3_ at costa is not bordered by a contiguous band of light scales

- *Sabatinca* sp. 46

1. Sc_2_ surrounded by darkest scales at costa
2. Rs_1_ surrounded/abutted by lightest scales at costa
3. Rs_2_ surrounded by darkest scales at costa
4. Rs_3_ surrounded by dark scales at costa

- *Sabatinca* sp. 47

1. Rs_3_ surrounded by medium scales at costa

- *Sabatinca delobeli*

1. Rs_2_ surrounded by darkest scales at costa

- *Sabatinca kristenseni*

1. Sc_2_ surrounded by darkest scales at costa
